# Membrane microdomains are crucial for *Mycobacterium marinum* EsxA-dependent membrane damage, escape to the cytosol, and infection

**DOI:** 10.1126/sciadv.ady0812

**Published:** 2026-01-01

**Authors:** Angélique Perret, Cristina Bosmani, Florence Leuba, Krikor Eblighatian, Aurélie Guého, Lyudmil Raykov, Nabil Hanna, Thierry Soldati

**Affiliations:** Départment de Biochimie, Faculté des Sciences, Université de Genève, Sciences II, 30 quai Ernest Ansermet, CH-1211 Geneva-4, Switzerland.

## Abstract

*Mycobacterium tuberculosis* disrupts the membrane of the *Mycobacterium*-containing vacuole (MCV) via its EsxA virulence factor, secreted via the ESX-1 system. We identified how pathogenic *Mycobacterium marinum* exploits host sterol-rich membrane microdomains to induce MCV damage during infection of *Dictyostelium discoideum* and murine microglial BV-2 cells. Transcriptomic and protein analyses revealed that *vacuolinC* is specifically induced in response to EsxA-mediated damage. Vacuolins initially associate with the MCV in a patchy distribution, coinciding with sterol-rich microdomain formation before entirely coating the MCV. Functional assays demonstrate that membrane microdomains potentiate l-leucyl–l-leucine methyl ester and EsxA-mediated membrane damage. Knockout of *vacuolins* and sterol depletion drastically reduce EsxA partitioning into membranes in vitro and decrease MCV damage and Mm escape to the cytosol, thereby substantially impairing Mm intracellular growth and phenocopying in Dd and microglial cells the attenuation of the Mm ΔRD1 mutant lacking ESX-1. Our results highlight host membrane microdomains as critical platforms exploited by virulent mycobacteria across evolutionary distant host phagocytes, thus representing potential therapeutic targets.

## Teaser

Sterol-rich microdomain accumulation at the MCV is crucial for Mm-induced damage and infection in *D. discoideum* and BV-2 cells.

## INTRODUCTION

Tuberculosis, caused by *Mycobacterium tuberculosis* (Mtb), is a major global health issue, killing 1.3 million people in 2022 (*1*). Alveolar macrophages are the first line of defense against Mtb in the lungs; however, Mtb manipulates their bactericidal phagosome maturation pathway to establish a replicative niche inside a modified phagosome, the *Mycobacterium*-containing vacuole (MCV) (*2*, *3*). *Mycobacterium marinum* (Mm), closely related to Mtb, causes a soft tissue disease in freshwater vertebrates with a similar cellular pathogenesis and infection course, which is notably the generation of granuloma, a hallmark of tuberculosis. Mm is widely used as a versatile experimental model for Mtb owing to its conserved virulence mechanisms, faster replication time, and easier laboratory manipulation (*4*, *5*).

*Dictyostelium discoideum* (Dd), a social amoeba, uses phagocytosis to feed on soil bacteria. Evolutionary conservation of the phagosome maturation pathway (*6*) and cell-autonomous defenses with animal innate immune phagocytes makes Dd a valuable model for studying host-pathogen interactions, particularly mycobacterial infections (*7*–*12*). After uptake, innocuous bacteria are enclosed in a phagosome that matures to expose them to acidic pH, hydrolases, reactive oxygen species, and toxic metals necessary for bacterial killing (*11*, *13*–*15*). In contrast, Mm and Mtb arrest phagosome maturation, generating an MCV from which they eventually escape in an ESX-1 (type VII secretion system)–dependent manner (*12*, *16*–*18*).

MCV membrane damage is crucial in the mycobacteria infection cycle. Mm-induced damage starts as early as 15 min postinfection, as one of the first signs of phagosome manipulation, leading to MCV tailoring, and largely depends on EsxA, a virulence factor secreted via ESX-1 (*8*, *19*). In both mammalian cells and Dd, the Endosomal Sorting Complexes Required for Transport (ESCRT) and autophagy machineries are recruited to the damage site via ubiquitination of the damaged membrane, acting as a “repair-me” signal (*12*). In Dd, TrafE, an E3 ubiquitin ligase, coordinates their recruitment, playing an important role in containing and restricting the infection (*20*, *21*). Damage accumulates later during infection, leading to bacterial escape to the cytosol and exposure to the xenophagy machinery (*19*–*22*). Mtb and Mm lacking the ESX-1 secretion system including EsxA (ΔRD1 mutants) induce less MCV damage, are contained, and therefore are strongly attenuated (*16*, *22*). Scattered in vitro evidence suggests that MCV membrane composition is crucial for EsxA activity and might be assisted by mycobacterial lipidic virulence factors such as phthiocerol dimycocerosates (PDIMs), which are complex branched cell wall–associated lipids that reportedly induce sterol clustering in host membranes (*23*–*25*).

In mammalian cells, the nature of the so-called “lipid rafts” has long been debated, but nowadays, the existence of membrane heterogeneities with sterol-rich microdomains is fully accepted. These membrane microdomains exist under two forms, the nonscaffolded microdomains and the more stable scaffolded microdomains. Flotillin-1 and -2 are evolutionary conserved microdomain stabilizers at the plasma membrane and phagosome (*26*–*29*). Microdomains are involved in infection by various pathogens, facilitating the activity of bacterial pore-forming toxins and cholesterol-dependent cytotoxins (*30*–*32*). Flotillins also play roles in infections by pathogens like *Leishmania* (*26*), *Chlamydia pneumoniae* (*33*, *34*), and *Anaplasma phagocytophilum* (*35*).

Dd vacuolins (VacA, B, and C) are functional flotillin homologs, capable of hetero-oligomerizing and behaving as integral membrane proteins (*36*–*38*). The absence of vacuolins impairs recognition and adhesion to particles, including Mm, affecting uptake but not killing of innocuous bacteria (*38*). In this study, we dissect the link between vacuolin/flotillin microdomains and the biogenesis, maintenance, and repair of the MCV during Mm infection in Dd and murine microglial BV-2 cells. We demonstrate that VacC is a specific reporter of infection with virulent Mm in Dd and that vacuolin/flotillin- and sterol-enriched membrane microdomains build up at the MCV in both host systems. Disruption of these microdomains through genetic manipulation of vacuolin/flotillins and/or biochemical sterol depletion increase resistance to Mm intracellular growth and to l-leucyl–l-leucine methyl ester (LLOMe)–induced sterile damage. Sterol-rich membrane microdomain components are required for EsxA membrane partitioning in vitro, MCV damage, and Mm cytosolic access in vivo in Dd and BV-2 cells.

## RESULTS

### *Vacuolin C* is specifically induced upon Mm infection

**Fig. 1.**
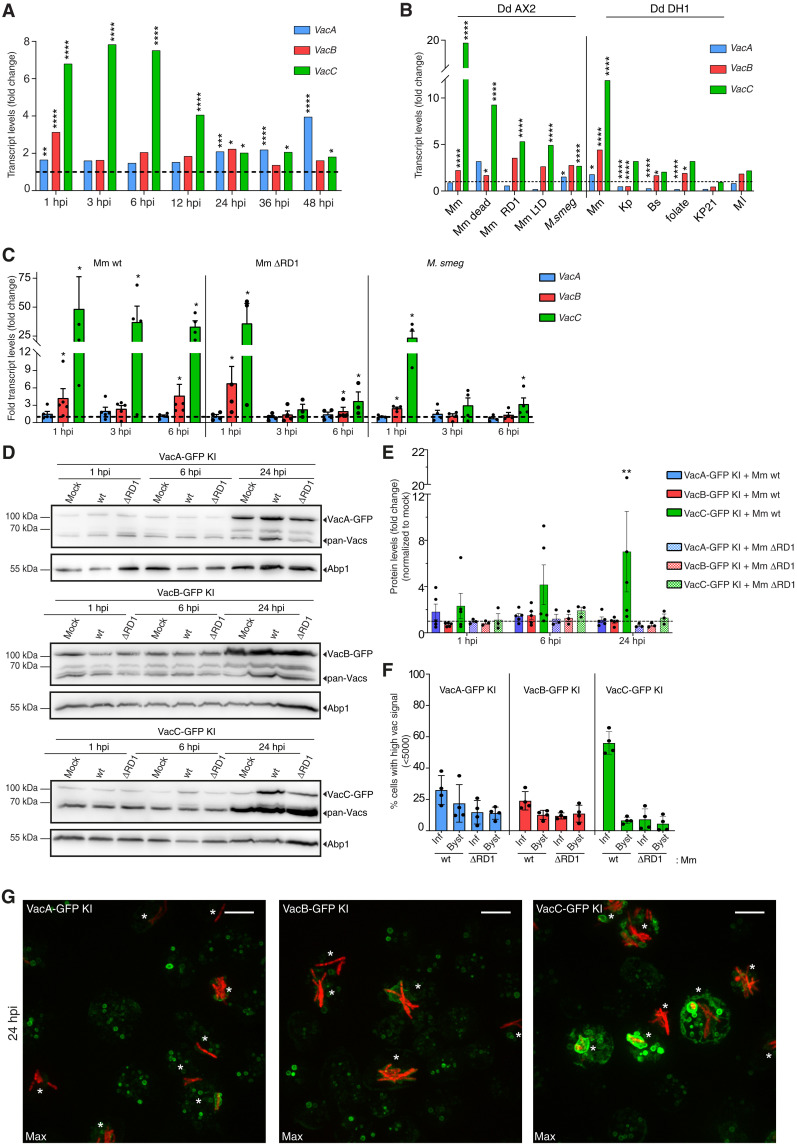
*vacC* is specifically induced upon Mm infection. (**A**) RNA-seq of FACS-sorted wt cells infected with GFP-expressing Mm at different hours postinfection (hpi). Fold change of each transcript compared to mock-infected cells (dashed line, *N* = 3, *P ≤ 0.05, **P ≤ 0.01, ***P ≤ 0.001, ****P ≤ 0.0001). (**B**) RNA-seq of AX2 or DH1 wt cells in contact with the indicated bacteria for 4 hours, normalized and compared to mock-infected cells (dashed line) (*N* = 3, *P ≤ 0.05, ****P ≤ 0.0001). Mm, Mm; *M. smeg*, *Mycobacterium smegmatis*; Kp, *Klebsiella pneumoniae*; Bs. *Bacillus subtilis*; Ml, *Micrococcus luteus*. (**C**) Quantitative RT-PCR of wt cell population infected with Mm wt or ΔRD1, or *M. smeg*. RNA levels were normalized to GAPDH and to mock-infected cells (dashed line, mean ± SEM, *N* = 4, *P ≤ 0.05, Mann-Whitney test). (**D**) Representative blots of lysates of a population of GFP knock-in (KI) cells infected with Mm wt or ΔRD1, or mock infected, immunoblotted with the indicated antibodies. (**E**) Quantification of (D) Vac-GFP bands normalized to Abp1 and mock infected cells (dashed line, N ≥ 3, **P ≤ 0.01, one-way ANOVA–Dunnett test). (**F**) Percentage of infected or bystander GFP KI cells with high-intensity (<5000) GFP signal at 24 hpi (mean ± SEM, *N* = 2, *n* ≥ 150 cells). (**G**) Representative Max projections of indicated KI cell lines infected with Mm wt expressing mCherry at 24 hpi; the same settings were used to image all cell lines. *, infected cells; scale bars, 10 μm.

To investigate whether mycobacteria manipulate *vacuolin* expression during infection, we analyzed the transcriptomic response of Dd wild-type (wt) Ax2(Ka) cells infected with green fluorescent protein (GFP)–expressing Mm wt as previously described (*39*). The *vacC* gene, poorly expressed in vegetative cells [Dicty express (*40*)], was significantly induced as early as 1 hour postinfection (hpi) and remained high throughout the 48-hour infection cycle (Fig. 1A). *VacB* was transiently highly induced at 1 hpi, while *vacA* was up-regulated at later time points (24 to 48 hpi).

To determine whether *vacC* induction was specific to mycobacteria, we analyzed the transcriptomic response of Dd Ax2(Ka) and DH1 wt cells in contact for 4 hours with various Gram-positive and Gram-negative bacteria and mycobacteria strains [Fig. 1B (*41*)]. *VacC* was significantly induced only when Dd was in contact with mycobacteria (Fig. 1B), with induction levels correlating with the strain’s pathogenicity and capacity to induce MCV damage. The highest induction was observed in contact with Mm wt and the lowest with nonpathogenic *Mycobacterium smegmatis*. RNA sequencing (RNA-seq) results were further validated by quantitative real-time polymerase chain reaction (qRT-PCR; Fig. 1C). The absolute fold change measured by qRT-PCR was higher than that observed by RNA-seq, likely due to low basal expression of *vacC*. Unlike the constant up-regulation of *vacC* with Mm wt infection, the Mm ΔRD1 mutant and *M. smegmatis* only transiently induced *vacC* expression at 1 hpi.

To determine whether VacC protein levels were similarly up-regulated upon Mm infection, endogenous protein levels of each vacuolin were assessed by Western blot using Vac-GFP knock-in strains [Vac-GFP KI (*38*)]. VacC-GFP accumulation was detectable at 6 hpi and significantly at 24 hpi compared to mock-infected cells (Fig. 1, D and E), while no significant accumulation was observed for VacA-GFP or VacB-GFP. In addition, infection with Mm wt, but not the Mm ΔRD1 mutant, induced high VacC-GFP levels (Fig. 1, D and E). Note that this analysis was performed on a mixed population of infected and bystander uninfected cells. In addition, detection of all three isoforms by the pan-vac antibody shows that VacA and VacB are more abundant than VacC as best visible in the VacC-GFP KI strain (Fig. 1D). High content (HC) microscopy investigations at the single-cell level confirmed higher VacC accumulation in Vac-GFP KI infected cells compared to bystanders (Fig. 1, F and G). In contrast, no significant difference in VacC-GFP levels was observed when cells were infected with the Mm ΔRD1 mutant. These results demonstrate that in Dd, VacC is a host reporter specific to Mm infection, likely triggered by EsxA-dependent MCV damage.

### Vacuolins gradually accumulate at the MCV throughout infection

**Fig. 2.**
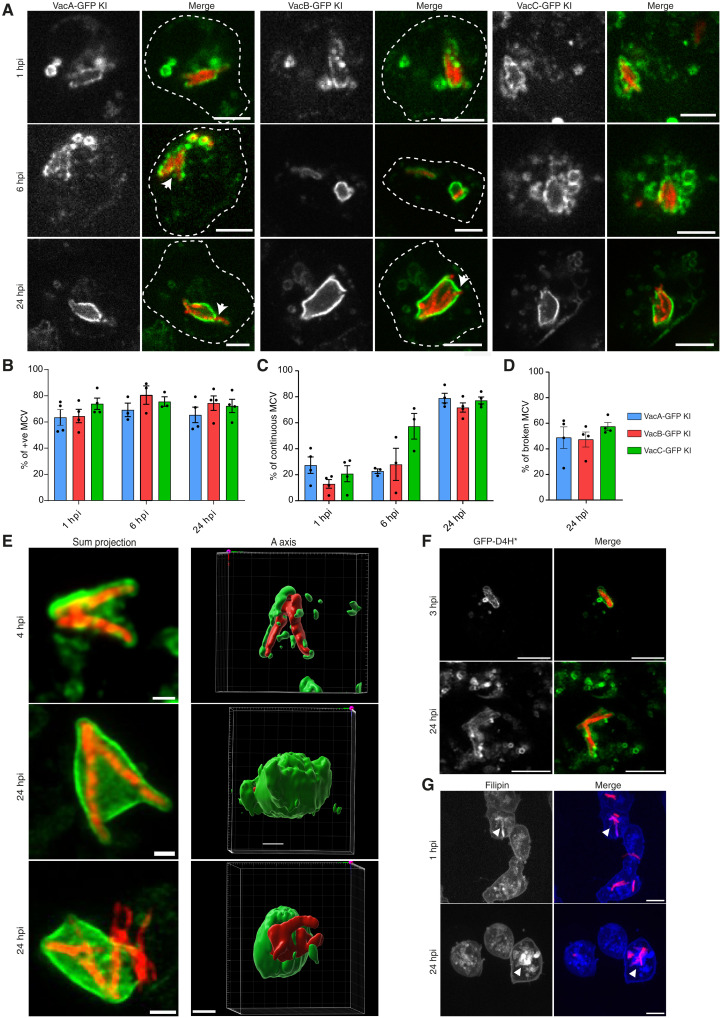
Vacuolin gradually accumulates at the MCV during infection. (**A**) Representative images of indicated Vac-GFP KI cell lines infected with Mm wt expressing mCherry. Arrows, broken MCVs; scale bars, 5 μm. (**B**) Quantification of (A) presenting the proportion of MCVs positive for each vacuolin (mean ± SEM, *N* = 4, *n* ≥ 200 MCVs). (**C**) Quantification of (A) presenting the percentage of MCVs showing a continuous vacuolin coat (mean ± SEM, *N* = 4, *n* ≥ 200 MCVs). (**D**) Quantification of the percentage of macroscopically broken MCVs at 24 hpi. (**E**) Representative live images of VacC-GFP KI cell infected with Mm wt expressing mCherry and 3D reconstruction; scale bars, 1 μm. (**F** and **G**) Representative images of wt cells infected with mCherry-expressing Mm wt fixed at indicated time points. (F) Cells expressing GFP-D4H* or (G) stained with filipin. Scale bars, 5 μm.

To investigate vacuolin trafficking in Dd, we used Vac-GFP KI cells infected with mCherry-expressing Mm and monitored vacuolin dynamics by live microscopy (Fig. 2, A and B). Each vacuolin was present at the MCV as early as 1 hpi, with about 60% of MCVs coated with a vacuolin on the first day of infection (Fig. 2B). The association of vacuolins with the MCV evolved over time from an initial patchy distribution pattern (Fig. 2, A, C, and E, top) and progressing to a continuous coat, with about 75% of vacuolin-positive MCVs displaying a complete vacuolin coat at 24 hpi (Fig. 2, C and E, middle). These results were confirmed using recombinant antibodies against endogenous VacA and VacB (fig. S1A).

To determine whether there are distinct MCV populations that are positive for a single vacuolin, we directly assessed the localization of pairs of vacuolin isoforms (fig. S1B), revealing that VacA and VacB always colocalize with VacC (fig. S1B), which arithmetically (Fig. 2B) implies that all three isoforms are present together and that there is no isoform-specific MCV.

At late stages of infection, MCV damage became macroscopically apparent, leaving only about 30 to 40% intact MCVs (Fig. 2D). The three-dimensional (3D) reconstruction of deconvoluted live images of VacC-GFP KI–infected cells provided a detailed view of the steps of vacuolin coat formation around the MCV (Fig. 2E). At 4 hpi, VacC-GFP formed patches around the MCV, which gradually expanded to fully enclose the MCV membrane at 24 hpi (Fig. 2E, top and middle). At this stage, Mm perforated both the MCV and its vacuolin coat to access the cytosol (Fig. 2E, bottom). To monitor sterol distribution at the MCV, we used filipin and the sterol sensor D4H* (*42*) (Fig. 2, F and G). While GFP-D4H* labeled the MCV membrane (Fig. 2F), filipin stained both the MCV membrane and the lumen (Fig. 2G). Both reporters colocalize at the MCV membrane throughout infection (fig. S1C). In addition, VacC-GFP and mScarlet-D4H* colocalized at the MCV, providing evidence for sterol enrichment (fig. S1D).

### Membrane microdomain components accumulate at the MCV

**Fig. 3.**
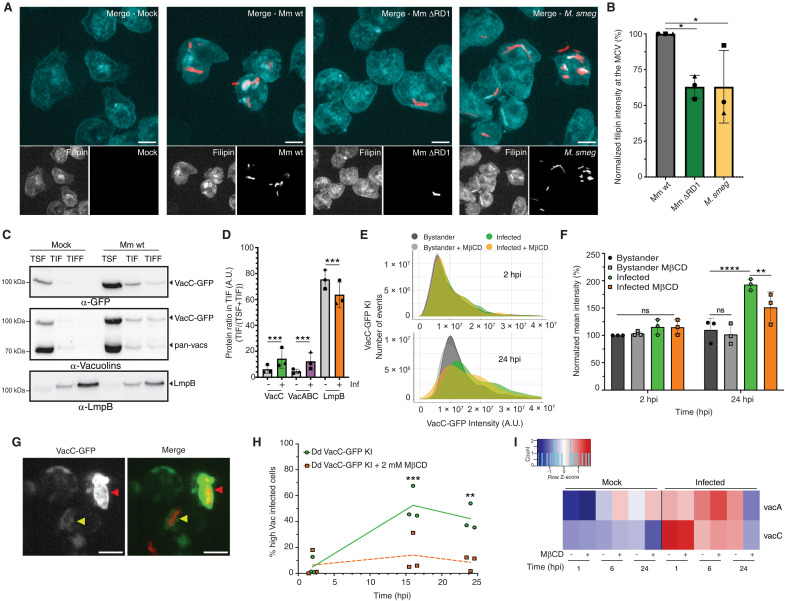
Membrane microdomain components accumulate at the MCV during infection in Dd. (**A**) Representative images of Dd wt cells infected with mCherry-expressing Mm wt, Mm ΔRD1, or *M. smegmatis* and fixed at 3 hpi. Scale bars, 5 μm. (**B**) Quantification of (A) representing the normalized filipin intensity at the MCV [mean ± SD, *n* = 3 (*n* cells ≥ 150); *N* = 3, *t* test with Welsch correction of paired comparison, *P ≤ 0.05]. (**C** and **D**) Dd VacC-GFP KI cells were mock infected or infected with Mm wt and, after 16 hpi, lysed in cold Triton X-100. The Triton soluble (TSF) and insoluble (TIF) fractions were recovered, as well as the floating fraction (TIFF) from a sucrose gradient. Equal protein amounts of each fraction were loaded and immunoblotted with the indicated antibodies. (C) Representative images of three independent experiments. (D) Quantification of (C) with the fraction of VacC-GFP (detected with the anti-GFP) and all three vacuolins (detected with the anti-vacuolins) in the TIF compared to the total (TSF + TIF) (mean ± SD, *n* = 1, *N* = 3, ***P ≤ 0.005, one-way ANOVA–Dunnett test). (**E** to **H**) Dd VacC-GFP KI cells infected with Mm wt expressing mCherry were monitored by HC microscopy. (E) Normalized distribution of total VacC-GFP intensity per cell during infection and treatment with 2 mM MβCD at 2 and 24 hpi (E) (*n* = 3; *N* = 3, *n* ≥ 50,000 cells). (F) Mean intensity of (E) (mean + SD, *n* = 3, *N* = 3, two-way ANOVA–Tukey test, **P ≤ 0.01, ****P ≤ 0.0001). [(G) and (H)] Analysis of (E) at the MCV level. (G) Representative image of MCV with high VacC-GFP intensity (red arrowhead) and low VacC-GFP intensity (yellow arrowhead). (H) Quantification of high VacC-GFP MCVs (GFP intensity <5000) after infection and treatment with MβCD [mean ± SEM, *n* = 3 (*n* cells ≥ 150); *N* = 3, cells, two-way ANOVA–Fisher’s LSD test, **P ≤ 0.01, ***P ≤ 0.005]. (**I**) RNA-seq analysis of infected wt cells untreated or treated with MβCD. Heatmap of vacuolin genes for mock and infected cells at 1, 6, and 24 hpi. *vacB* was filtered out for low read counts.

Quantification of the filipin staining intensity at MCVs containing various mycobacteria revealed that the highest accumulation occurs around Mm wt compared to the attenuated Mm ΔRD1 mutant and the nonpathogenic *M. smegmatis* (Fig. 3, A and B).

Because of this increase of sterol content in the MCV, and in analogy to flotillins, we hypothesized that vacuolins might be part of sterol-rich detergent-resistant membranes. To test this, an identical number of Vac-GFP KI (fig. S2A) or Vac-overexpressing cells (OE; fig. S2B) were lysed in cold Triton X-100, and Triton-soluble and -insoluble fractions (TSF and TIF, respectively) were separated by centrifugation. Then, TIF was floated by centrifugation on a step sucrose gradient [Triton-insoluble floating fraction (TIFF)]. LmpB, a CDC36-like scavenger receptor that partitions in the detergent-resistant fraction of the plasma membrane (*43*), was used as a reference for the fractionation. As shown in Fig. 3D, the amount of LmpB collected in TIF appeared slightly lower in infected cells. Under basal conditions, only a small fraction of each vacuolin isoform, either as a GFP-tagged or endogenous version detected by a pan-Vac antibody, was present in TIF (fig. S2, A and B). Overexpression increased partitioning in TIF (fig. S2B). Infection with Mm wt resulted in a larger fraction of VacC-GFP and endogenous vacuolins, as quantitated with the pan-vac antibody, partitioned into TIF, with a twofold enrichment at 18 hpi (Fig. 3, C and D). In addition, extraction of sterols with methyl-beta-cyclodextrin (MβCD) strongly decreased vacuolin accumulation during infection (Fig. 3, E to H, and fig. S2, C to H). Quantitative imaging showed a twofold increase in VacC-GFP signal in infected cells at 24 hpi, which was abolished by MβCD treatment (Fig. 3, E and F). Analysis at the MCV level revealed an 80% decrease of VacC-GFP accumulation at the MCV after 16 hours of MβCD treatment (Fig. 3, G and H). A similar trend was observed for VacA-GFP (fig. S2, C to E) and VacB-GFP (fig. S2, F to H). Moreover, RNA-seq investigation of the impact of MβCD treatment on *vacuolin* expression confirmed that *vacA* and *vacC* are induced during infection by Mm wt and that, at 24 hpi, MβCD treatment prevents this up-regulation (Fig. 3I). Overall, these results demonstrate that throughout the infection cycle in Dd, the MCV gradually accumulate sterols and vacuolins, two components of membrane microdomains.

### Microdomain components localize at the MCV in murine microglial BV-2 cells

**Fig. 4.**
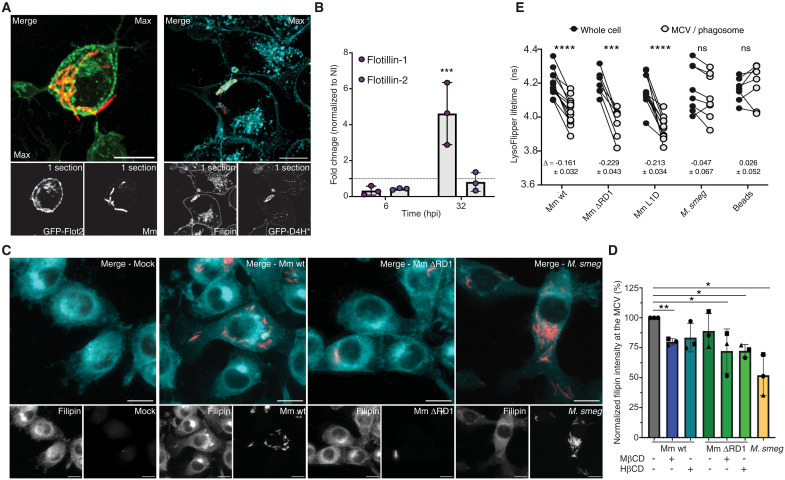
Sterol-rich microdomain components accumulates at the MCV in BV-2 cells. (**A**) BV-2 cells expressing Flot2-GFP or GFP-D4H* were infected with mCherry-expressing Mm wt, fixed at 24 hpi and stained with filipin. Scale bars, 10 μm. (**B**) Quantitative RT-PCR of infected BV-2 cells with Mm wt. RNA levels were normalized to GAPDH and to mock cells (dashed line, mean ± SD, *N* = 3, *P ≤ 0.05, Mann-Whitney test). (**C** and **D**) BV-2 cells were infected with GFP or mCherry-expressing Mm wt, Mm ΔRD1 or *M. smegmatis*, fixed at 5 hpi and stained with filipin. (C) Representative images. Scale bars, 10 μm. (D) Quantification of (C) representing the normalized filipin intensity at the MCV. [mean ± SD, *n* = 3 (*n* cells ≥ 150); *N* = 3, *t* test with Welsch correction of paired comparison, *P ≤ 0.05]. (**E**) BV-2 cells were infected and incubated with LysoFlipper at 6 hpi. Lifetime of the LysoFlipper probe was measured at the MCV and on the whole cell (paired *t* test, *P ≤ 0.05, **P ≤ 0.01, ***P ≤ 0.005, ****P ≤ 0.0001).

To directly probe whether the presence of microdomain components at the MCV is conserved in animal phagocytes, we used murine microglial BV-2 cells that are adherent, motile, and constitutively phagocytic (*44*). We have previously used these microglial cells in parallel with the Dd-Mm model to perform a genome-wide fitness analysis that revealed strong conservation of genetic requirements for virulence and survival in evolutionarily distant phagocytes (*45*) as well as to validate anti-infective compounds primarily identified using the Dd-Mm platform (*46*). We monitored the localization of the microdomain organizer flotillins and sterols using BV-2 cells stably expressing Flot2-GFP or the genetically encoded sterol probe GFP-D4H*. Flot2 and D4H* localized at the MCV at 24 hpi (Fig. 4A), suggesting, like in Dd, an enrichment of sterol-rich microdomains. In addition, filipin staining of infected GFP-D4H* BV-2 cells confirmed the presence of sterols at the MCV membrane and in its lumen (Fig. 4A). By performing qRT-PCR on BV-2 cells infected with Mm wt, we detected a higher level of Flot-1 mRNA at 32 hpi (Fig. 4B), reminiscent of the *vacC* reporter in the Dd model.

To confirm that sterol accumulation at the MCV of Mm wt is one of the dominant changes, we quantified the filipin staining intensity at the MCV of Mm wt compared to Mm ΔRD1 and *M. smegmatis* by HC microscopy (Fig. 4, C and D), as performed in Dd (Fig. 3, A and B). In addition, treatment with MβCD or 2-hydroxypropyl-β-cyclodextrin (HβCD) during infection strongly decreased filipin intensity at the MCV, confirming in BV-2 cells that cyclodextrin depletes MCV sterols (Fig. 4D). The MCV containing *M. smegmatis*, a nonpathogenic environmental *Mycobacterium* that does not secrete the membrane damaging EsxA, did not accumulate as much sterol (Fig. 4, C and D).

To monitor the impact of the changes in composition of the MCV membrane on its biophysical characteristics, we used the LysoFlipper probe (*47*) that accumulates in the endolysosomal system of animal cells, and the fluorescence lifetime (τ) of which informs about a combination of local membrane tension, lipid packing, and overall membrane composition. Because of the nature of our experiments, the fluorescence lifetime of LysoFlipper reports the steady-state membrane characteristics and not local transient variations in membrane tension, such as those arising from membrane damage. The LysoFlipper lifetime did not differ between the membrane of a bead-containing phagosome and the rest of the endolysosomal membranes throughout the cell (Fig. 4E). τ values around 4.2 ns are lower than those reported for plasma membranes of various mammalian cells (*47*) and correspond to measured endolysosomal compartments (*47*). In sharp contrast, the MCV of Mm wt, but also of Mm ΔRD1, and Mm L1D exhibited strongly and significantly decreased τ compared to endolysosomes, with Δτ in the range of −0.2 (Fig. 4E). This indicates that the changes observed at the MCV are specific to vacuoles containing mycobacteria but do not correlate with membrane damage inflicted by the mycobacteria. In addition, the *M. smegmatis*–containing MCV exhibited an intermediate but nonsignificant Δτ in the range of −0.05, paralleling the lower sterol accumulation at that MCV. The decreased τ values at MCVs indicate that these membranes have lipid packing defects probably as a result of increased sterol content.

Altogether, the data indicate that the MCV composition in BV-2, like in Dd, is drastically different from the rest of the cellular compartments and accumulates sterols and microdomain organizers. It can be expected that the lipid packing defects resulting from high sterol concentrations can fragilize the MCV membrane and render it more susceptible to EsxA damaging activity.

### Disruption of membrane microdomains confers resistance to Mm infection

**Fig. 5.**
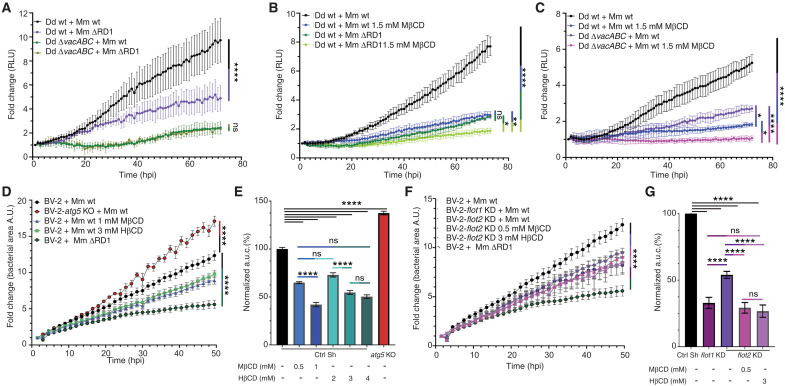
Disruption of membrane microdomains confers resistance to infection. (**A** to **C**) Dd wt and Δ*vacABC* cells were infected with bioluminescent Mm wt or Mm ΔRD1, and nontreated or treated with 1.5 mM MβCD. Bioluminescence was measured for 72 hours (mean fold change ± SEM, *N* = 3, two-way ANOVA–Fisher’s LSD test, *P ≤ 0.05, **P ≤ 0.01, ****P ≤ 0.0001). RLU, relative luminescence unit. (**D** to **G**) BV-2 cells were infected with GFP-expressing Mm wt and monitored by HC microscopy for 48 hours. [(D) and (F)] Growth curves of Mm measured as the area sum of the GFP signal at every time point (mean fold change ± SEM, *n* = 3; *N* = 3, two-way ANOVA–Fisher’s LSD, ****P ≤ 0.0001). RFU, relative fluorescence unit. [(E) and (G)] Analysis of the area under the curve of (D) and (F) normalized to Mm wt (100%) and Mm ΔRD1 (0%) (mean ± SD, *n* = 3; *N* = 3, one-way ANOVA–Dunnett test, ****P ≤ 0.0001).

To test whether manipulation of microdomain components affects Mm virulence, we monitored the growth of wt and vacuolin mutant Dd strains on bacterial lawns containing mycobacteria. Counterintuitively, whereas Dd wt does not grow well in the presence of Mm wt, cells with KOs of one or more vacuolins grew up to 10-fold better (fig. S3, A and B), while growth in the presence of Mm ΔRD1 was unaffected. To directly examine the impact of *vacuolin* KO and sterol depletion, we measured intracellular Mm growth by monitoring either lux-expressing Mm in a plate reader (Fig. 5, A to C) or GFP-expressing Mm by flow cytometry (fig. S4, E and F). In the absence of two (Δ*vacBC*) or all three vacuolins (Δ*vacABC*), Mm wt was not able to grow as efficiently as in Dd wt cells, with a reduction of 50% (Fig. 5, A and C) to 80% (fig. S3E) at 72 hpi. Intracellular growth of the attenuated strain Mm ΔRD1, which induces only very limited MCV damage, is not significantly affected by *vacuolin* KO (Fig. 5A).

In Dd, sterol depletion with MβCD decreased Mm wt growth (Fig. 5B) in a dose-dependent manner (fig. S3C), reaching a reduction similar to the low growth level of the attenuated Mm ΔRD1 (Fig. 5B and fig. S3C). These doses of MβCD do not affect Mm growth in medium (fig. S3D). Similar to the low impact of the absence of vacuolins, MβCD treatment only weakly affected the intracellular growth of Mm ΔRD1 (Fig. 5B and fig. S3C). Last, sterol depletion in Δ*vacABC* cells produced a significant additive inhibition of intracellular growth of Mm wt (Fig. 5C). Our data indicate that vacuolins and sterols are crucial susceptibility factors, as their absence or depletion confers resistance to infection, and the combination abolishes Mm growth.

To gain further insight into the resistance mechanism, we investigated the dynamics of infection at the single-cell level using Fluorescence-activated cell sorting (FACS) and automated HC microscopy. In a standard infection, the proportion of infected cells decreases passively with time because they grow slower than uninfected bystander cells, and infection dissemination does not compensate. The doubling time of Mm is similar to that of the Dd host, both being around 8 hours (*48*, *49*). The percentage of infected Δ*vacBC* cells decreased faster than wt cells, down to two-thirds compared to wt at 21 hpi (fig. S3F), indicating active and early “curing” of the infection. This curing might be due to early pathogen release from the host cell, either by host cell death, exocytosis, or nonlytic egress termed ejection (*18*, *50*). To investigate whether interference with vacuolin microdomains is involved in early release, HC microscopy quantification of the proportion of extracellular versus intracellular Mm was performed in Dd wt and Δ*vacABC* cells (fig. S3G). Release of Mm wt from Dd wt and Δ*vacABC* cells was observed with the same kinetics, with 50% of bacteria found extracellular at 27 hpi (fig. S4G). These data suggest that early release is not a major mechanism to explain the resistance of *vacuolin* KO mutants to Mm wt infection, but likely containment and restriction are.

To monitor the evolutionary conservation of the involvement of microdomain components in infection, we quantified intracellular Mm growth in BV-2 cells by HC microscopy. Mm wt exhibited enhanced growth in *atg5* KO cells, confirming that, similar to Dd, xenophagy is the major restriction pathway (*51*, *52*) (Fig. 5, D and E). In addition, again, like in Dd, the intracellular growth of Mm ΔRD1 is strongly attenuated (Fig. 5, D to G). Treatment with increasing doses of MβCD or HβCD also resulted in a dose-dependent decrease of intracellular Mm growth (Fig. 5, D and E). We generated stable BV-2 cell lines with knockdown (KD) of *flot-1* or *flot-2* (fig. S3H), the vacuolin orthologues, and monitored Mm intracellular growth (Fig. 5, F and G). Compared to the control (Ctrl Sh), KD of *flot-1* or *flot-2* reduced Mm intracellular growth (Fig. 5, F and G), similar to the decrease observed with CD treatments. Sterol depletion of *flot-2* KD cells also resulted in an additive effect with an even stronger attenuation of Mm wt growth (Fig. 5, F and G). Altogether, these data show that sterol-rich microdomains are important susceptibility factors for Mm infection in Dd and BV-2 cells.

In both host systems, EsxA-dependent damage to the MCV membrane, eventually resulting in escape of Mm to the cytosol, is a prerequisite for efficient infection (*12*, *18*, *20*). In that context and paradoxically, both increased containment in the MCV and precocious escape to the cytosol followed by xenophagy can result in reduced bacterial loads (fig. S4). For example, the Mm ΔRD1 mutant is much weaker at both inducing damage and escaping from its compartment, causing a strong attenuation by confinement, possibly because of limited nutrient access (*19*) (fig. S4B1). Apparent attenuation can also be caused by host restriction. In cells with impaired MCV damage repair, Mm wt escapes precociously to the cytosol and is rapidly recaptured and restricted by xenophagy (fig. S4B2) (*19*–*21*). We hypothesized that vacuolin/flotillin microdomains modulate Mm escape from or retention inside the MCV by affecting the level of damage induced to the MCV membrane.

### *Vacuolin* KOs and MβCD treatment dampens the transcriptomic response to infection-induced damage

We previously used time-resolved RNA-seq analysis to monitor transcriptomic reactions and adaptations to an infection with Mm wt, revealing a strong impact on lipid metabolism, cell cycle, stress responses, and reactive oxygen species (ROS) production and including a major stimulation of both the ESCRT and autophagy pathways (*39*). As confirmed by qRT-PCR on selected transcripts, the latter two pathways are strongly up-regulated in response to Mm wt-induced MCV damage and not during infection with Mm ΔRD1 (*19*). The two pathways serve a crucial role in membrane repair, Mm containment, and restriction of bacteria that gain access to the cytosol (*20*, *21*). It is important to note that these earlier studies revealed that the ESCRT and autophagy pathways not only are intervening physically and transiently at various stages of infection but also are regulated at the transcriptional level, likely to support a sustained response to infection (*19*–*21*).

**Fig. 6.**
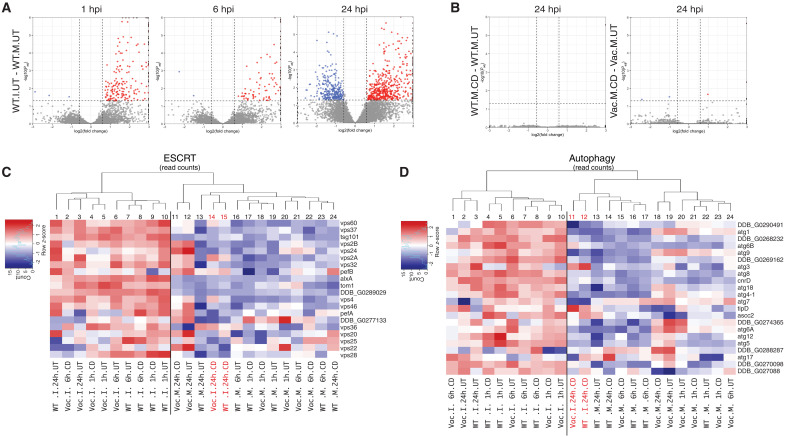
Cyclodextrin treatment dampens the damage/repair response during infection. (**A** to **D**) RNA-seq analysis of mock and infected Dd wt cells compared to Δ*vacABC* and MβCD-treated cells. [(A) and (B)] Volcano plots of selected comparisons after performing DEG analysis. Selected cutoff for statistical significance is abs(log2FC) > 0.5848 and *P*_adj_ <0.05. Blue dots represent down-regulated genes, and red dots represent up-regulated genes. (A) Volcano plots of untreated infected Dd wt cells compared to untreated mock-infected Dd wt cells at the indicated time point postinfection. (B) Volcano plots of mock-infected Dd wt and Δ*vacABC* cells treated with 2 mM on MβCD compared to the respective untreated condition at 24 hpi. [(C) and (D)] Heatmaps of normalized read counts. Filtered and normalized read counts were interrogated for previously defined lists of genes of interest and clustered by conditions. This included a gene set associated to the ESCRT machinery (C) and the autophagy pathway (D). WT, Dd wt; Vac, Dd Δ*vacABC*; M, mock; I, infected; UT, untreated; CD, MβCD.

In the present study, to uncover a potential signature for the absence of vacuolins and for MβCD treatment during infection, we performed similar RNA-seq experiments (Fig. 6 and fig. S5 and https://zenodo.org/records/15111828). As an overview, the volcano plots confirm that the dominant up and down modulations occur as a function of the infection status in Dd wt and are visible as early as 1 hpi and increase until 24 hpi (Fig. 6A and fig. S5B). The volcano plots of the binary comparisons visually support that MβCD treatment of mock-infected wt or Δ*vacABC* cells did not have a significant effect on their transcriptomes (Fig. 6B and fig. S5, A and E). Notably, at 24 hpi, treatment with MβCD dampens the transcriptomic response induced by the infection in Dd wt and Δ*vacABC* cells (fig. S5, C and H).

Deeper analyses using clustered heatmaps based on read counts for genes related to the ESCRT and autophagy pathways confirmed a strong up-regulation of most of the interrogated genes in response to Mm wt infection and showed a distinct clustering of the infected conditions, clearly separating infected samples from mock-infected ones (Fig. 4, C and D). The clustering analysis also revealed a notable effect of MβCD treatment. While all the infected samples cluster together (columns 1 to 10) (Fig. 4, C and D), the infected samples treated with MβCD at 24 hpi do not cluster with the other infected conditions. Instead, they cluster together with the mock-infected conditions (columns 11 to 24). These changes indicate that the infected MβCD-treated cells exhibit a transcriptomic profile more similar to mock-infected cells than to untreated infected ones, suggesting that MβCD strongly dampens the infection- and damage-driven transcriptional program.

These results demonstrate that MβCD treatment and, to a lesser extent, absence of vacuolins have a suppressive effect on the transcriptome changes affecting the repair-related genes in response to infection-induced damage, very likely because both conditions reduce Mm-induced MCV damage, thereby diminishing the need for a strong and prolonged activation of the ESCRT and autophagy pathway-related genes.

### Manipulation of membrane microdomain components increases resistance to LLOMe-induced endolysosomal damage

**Fig. 7.**
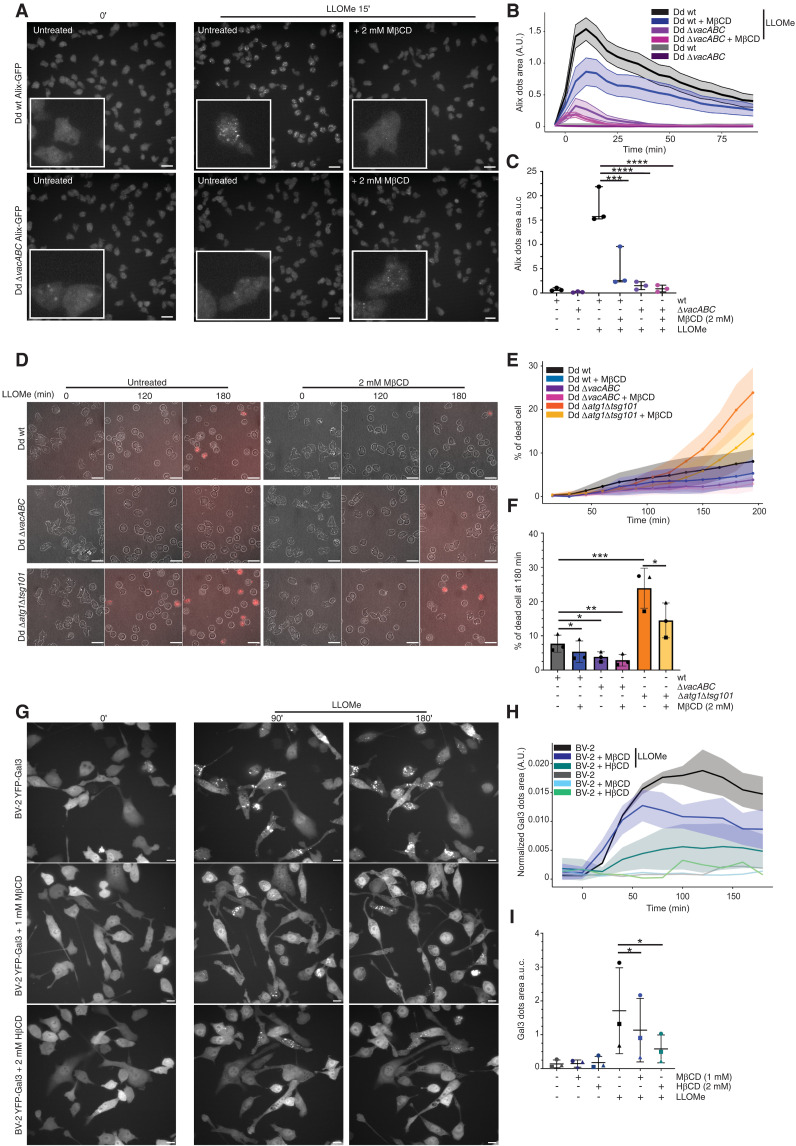
Disruption of membrane microdomains renders cells more resistant to LLOMe activity. (**A** to **C**) Dd wt and Δ*vacABC* cells expressing Alix-GFP, treated or not with 2 mM of MβCD, were submitted to 4.5 mM LLOMe. (A) Representative live images of cells before treatment and 25 min after LLOMe addition; insets are magnified 3.5-fold. Scale bars, 5 μm. (B) One representative experiment showing the area of Alix dots through time. The analysis is running per cell with <600 cells per condition. (C) Area under the curve of (B) (mean ± SD, *n* = 3; *N* = 3, one-way ANOVA–Dunnett test, ***P ≤ 0.005, ****P ≤ 0.0001). (**D** to **F**) Dd wt, Δ*vacABC*, and Δ*atg1*Δ*tsg101* treated or not with 2 mM of MβCD were submitted to 10 mM LLOMe. (D) Representative live images of cells during the assay. Scale bars, 5 μm. (E) Dynamics of cell death through time. The analysis is running per cell with <600 cells per condition. (F) Quantification of the percentage of dead cells at 180 min posttreatment (mean ± SD, *n* = 3; *N* = 3, *t* test with Welsch correction of paired comparison, *P ≤ 0.05, **P ≤ 0.01, ***P ≤ 0.005, ****P ≤ 0.0001). (**G** to **I**) BV-2 expressing YFP-Gal3 treated or not with 1 mM MβCD or 2 mM HβCD were submitted to 0.45 mM LLOMe. (G) Representative live images of cells during the assay. Scale bars, 10 μm. (H) One representative experiment showing the normalized area of Gal3 dots (Gal3 area/cell area) through time. (I) Area under the curve of (H) (mean ± SD, *n* = 3; *N* = 3, one-way ANOVA–Dunnett test, *P ≤ 0.05).

We and others have shown that LLOMe, an endolysosome membrane disrupter, phenocopies several features of EsxA-induced MCV damage (*21*, *53*, *54*). While mechanistically distinct from EsxA activity, LLOMe provides a robust tool to assess the general contribution of microdomain components to the structural integrity and repair capacity of the endolysosomal compartment by inducing reversible synchronous and homogenous damage. Using LysoSensor, a pH-dependent chemical probe accumulating in acidic compartments, we followed damage induced by LLOMe treatment (fig. S6A). Dd wt cells lost LysoSensor signal faster and to a higher extent after LLOMe addition than Dd Δ*vacABC* cells and recovery to the initial signal was delayed (30 min versus 15 min), indicating a decrease in damage induced by LLOMe in Dd Δ*vacABC* cells (fig. S6A). In addition, we monitored the recruitment of reporters of the ESCRT-mediated membrane-repair machinery (Fig. 7 and fig. S6B). Dd wt and *vacuolin* KO cells stably expressing Alix-GFP or GFP-Vps32 were pretreated or not with 2 mM MβCD and imaged by live HC microscopy. Before the addition of LLOMe, all cells had a similar cytosolic signal (Fig. 7A and fig. S6B). Instantaneously after LLOMe addition, puncta formed at sites of ESCRT-mediated repair, peaking at around 5 to 10 min for Alix-GFP (Fig. 7B) and at around 15 to 20 min for GFP-Vps32 (fig. S6C). This timing agrees with their sequential roles in ESCRT recruitment and repair activity (*55*). The LLOMe effect is transient, because cells fully repair and recover. Both Dd Δ*vacBC* and Δ*vacABC* cells, as well as cells treated with MβCD, showed reduced recruitment of ESCRT components and a faster disappearance of these structures, indicating a faster recovery (Fig. 7B and fig. S6C). Quantitation of the overall extent of reporter recruitment indicated that the basal level of Alix-GFP and GFP-Vps32–positive structures is low and is maximally stimulated by LLOMe in wt cells (Fig. 7C and fig. S6D). The reduction of both reporters in Dd Δ*vacBC* and Δ*vacABC* cells is around three- to fivefold, values indistinguishable from those observed in wt cells treated with MβCD. In addition, MβCD treatment of *vacuolin* KO cells further significantly reduced recruitment down to basal levels (Fig. 7C and fig. S6D).

Inhibition of repair leads to a higher cell susceptibility to extensive LLOMe-induced membrane damage, eventually leading to cell death (Fig. 7, D to F). As expected, inhibition of ESCRT and autophagy machineries in the Dd Δ*atg1*Δ*tsg10* cell line led to a higher susceptibility to LLOMe compared to Dd wt cells (Fig. 7, D to F). The viability of Dd Δ*vacABC* cells upon LLOMe treatment was better than that of Dd wt cells. Moreover, sterol depletion with MβCD increased the viability of every cell line, including the Dd Δ*atg1*Δ*tsg10* that is strongly impaired in membrane repair. The latter observation is strong evidence that MβCD decreases membrane damage as opposed to improve repair (Fig. 7, D to F). In addition, we also interpret the decrease in recruitment of the ESCRT machinery and the maintenance of an intact cell morphology as reflecting a strong reduction in LLOMe-induced damage resulting in increased cell viability, contrary to what would happen if repair was inhibited (*21*).

The higher resistance to membrane damage upon LLOMe treatment was also validated in BV-2 cells expressing YFP-Gal3 (Fig. 7, G to I). Immediately after the addition of LLOMe, YFP-Gal3 puncta form at the damage sites. This reaction was dampened by cyclodextrin treatment, with a higher protective effect observed for HβCD, with a twofold decrease of the YFP-Gal3 dots area (Fig. 7, H and I). Similar to Dd, quantitation of the overall extent of YFP-Gal3 recruitment indicated that the basal level of YFP-Gal3–positive structures is low and is maximally stimulated by LLOMe (Fig. 7I).

To preclude that CD impairs lysosome function, which could result in limited LLOMe activation by protease cleavage, we tested whether the compartments were still acidic and proteolytic (fig. S6, E to L). As a reference in Dd, we used a ΔPIKfyve cell line, known for its defective endolysosomal acidification and digestive capacity (*56*). In Dd and BV-2 cells treated with CD, the pinocytic compartments were acidic as measured with lysosensor (fig. S6, E and F) and Lysotracker red (LTR; fig. S6, I and J), respectively. The proteolytic activity, measured as the dequenching of DQgreen-BSA, was also not significantly affected (fig. S6, G and H, and K and L). In conclusion, manipulation of membrane microdomain components renders the endolysosomal compartment more resistant to damage induced by the lysosomotropic membrane disrupter LLOMe.

### Microdomain disruption impairs Mm membranolytic activity in vivo

**Fig. 8.**
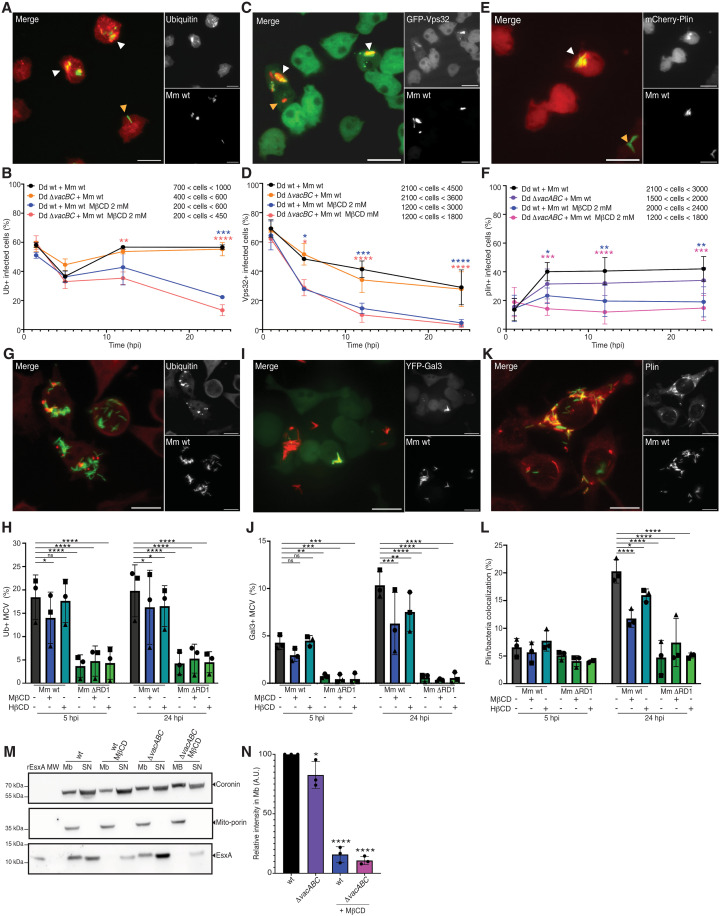
Microdomain components are important for Mm–induced MCV damage and EsxA membranolytic activity. (**A** to **F**) Dd wt, Δ*vacBC*, and/or Δ*vacABC* cells were infected with GFP- or mCherry-expressing Mm wt, treated or not with 2 mM MβCD, and, at the indicated time points, either immunostained for ubiquitin (FK2) or imaged live by HC microscopy. [(A), (C), and (E)] Representative images. Scale bars, 5 μm. [(B), (D), and (F)] Quantification of infected cells with reporter labeling (mean ± SD, *N* = 3, two-way ANOVA–Fisher’s LSD test, *P ≤ 0.05, **P ≤ 0.01, ***P ≤ 0.005, ****P ≤ 0.0001). (**G** to **L**) BV-2 wt cells or expressing YFP-Gal3 were infected with GFP- or mCherry-expressing Mm wt, treated or not with 1 mM MβCD or 2 mM HβCD, and, at the indicated time points, either fixed and immunostained for ubiquitin (FK2) or Plin, or imaged live by HC microscopy. [(G), (I) and (K)] Representative images. Scale bars, 10 μm. (H) Quantification of (G) with the percentage of MCV with ubiquitin labeling. (J) Quantification of (I) with the percentage of MCV with Gal3 labeling. (L) Quantification of (K) with the percentage Plin/Mm colocalization (mean ± SD, *n* = 3; *N* = 3, two-way ANOVA–Fisher’s LSD test, *P ≤ 0.05, **P ≤ 0.01, ***P ≤ 0.005, ****P ≤ 0.0001). (**M** and **N**) Recombinant EsxA (rESAT-6) was incubated with postnuclear supernatant (PNS) of wt or Δ*vacABC* cells, pretreated or not with 10 mM MβCD, followed by separation into supernatant (SN-cytosol) and pellet (Mb-membrane). Identical protein amounts were loaded and immunoblotted with the indicated antibodies. (M) Representative Western blot of three independent experiments. (N) Quantification of the fraction of EsxA in the membrane fraction normalized to mitochondrial porin (one-way ANOVA–Dunnett test, mean ± SD, *N* = 3, *P ≤ 0.05, ****P ≤ 0.0001).

Given the above conclusion, we wondered whether sterol-rich microdomains are necessary for EsxA activity during infection. In Dd, ubiquitination of MCV and Mm surface proteins by the E3 ubiquitin ligase TrafE is one of the earliest hallmarks of damage at the MCV and is essential to trigger the recruitment of both the ESCRT and the autophagy machineries (*20*, *21*). Once Mm reaches the cytosol, its surface becomes coated with the lipid droplet protein perilipin [mCherry-Plin (*57*)]. To monitor the steps of Mm-induced MCV damage and cytosol escape, we first quantitated ubiquitin labeling and GFP-Vps32 recruitment at MCVs in wt and *vacuolin* KO cells, with and without MβCD treatment (Fig. 8, A to F). In Dd wt and Δ*vacBC* cells, ubiquitin was found early around 60% of Mm, whereas it decreased to around 40% at 5 hpi before increasing to around 60%. Treatment with MβCD significantly decreased ubiquitination to approximately 20% at 24 hpi (Fig. 8, A and B). The early presence and subsequent decrease in GFP-Vps32 association with the MCV in Dd wt and Δ*vacBC* cells followed a similar pattern to that of ubiquitin and again were drastically reduced in MβCD-treated cells, with 5% of infected cells showing Vps32 recruitment at 24 hpi compared to about 30% in nontreated cells (Fig. 8, C and D). In contrast to these reporters of damage and repair, translocation to the cytosol, monitored by Plin association with the Mm surface, was reduced under all conditions compared to wt cells (Fig. 8, E and F). At 1 hpi, only a minor fraction of Mm is exposed to the cytosol (around 15%). From 5 hpi onward, Mm was increasingly associated with mCherry-Plin, reaching a plateau in Dd wt and Δ*vacABC* cells around 45 and 35%, respectively (Fig. 8, E and F). Although this apparent difference falls below the significance threshold, the trend was consistently observed across all replicates (fig. S7A). Notably, MβCD treatment decreased association with Plin in both cell types, with a plateau around 15 to 20% (Fig. 8, E and F). Together, these results indicate that in Dd, disturbing membrane microdomains by knockout of vacuolins and sterol depletion limits damage to the MCV and severely inhibits cytosol translocation of Mm.

As sterol depletion with CD similarly decreases Mm intracellular growth in BV-2 cells and protects against LLOMe-induced damage, we assessed whether, similar to Dd cells, CD treatment reduces Mm-induced damage and cytosol access. First, CD treatment appeared to constrain the spreading of Mm wt inside BV-2 cells as revealed by an increase of the area of microcolonies, which we interpret as indicative of confinement in the MCV, similar to that of the Mm ΔRD1 mutant (fig. S8, A to G). In addition, CD treatment increased microcolony area in a dose-dependent manner and to a similar extent as *flot-1* or *flot-2* KD. Again, sterol depletion of *flot-2* KD revealed an additive effect on confinement (fig. S9, C, D, and G).

Similar to Dd, we quantitated the ubiquitination and YFP-Gal3 recruitment at the MCV, two early signs of membrane damage in BV-2 cells (Fig. 8, G to L). Ubiquitin was found early around 18% of Mm wt at 5 hpi and remains stable at 24 hpi, whereas it was found to mark only about 5% of the Mm ΔRD1 MCV at 5 and 24 hpi (Fig. 8, G and H). CD treatment significatively decreased ubiquitination of the Mm wt MCV at 5 and 24 hpi but did not affect the Mm ΔRD1 MCV (Fig. 8, G and H). YFP-Gal3 was found around 5% of the Mm wt MCV at 5 hpi and increased to more than 10% at 24 hpi but remained weakly associated with the Mm ΔRD1 MCV from 5 to 24 hpi (Fig. 8, I and J). Treatment with CD significantly decreased YFP-Gal3 recruitment to approximately 7% at 24 hpi (Fig. 8, I and J). Mm translocation to the cytosol was monitored by quantitating Mm association and colocalization with Plin (Fig. 8, K and L). As expected, only 5 to 7% of Mm wt and ΔRD1 were coated by Plin at 5 hpi, and this percentage increased to 20% for Mm wt at 24 hpi but remained low for Mm ΔRD1(Fig. 8, K and L). Similar to ubiquitin and YFP-Gal3, Plin association with Mm wt was significantly decreased upon CD treatment with an almost twofold decrease for MβCD (Fig. 8, K and L). Our results in BV-2 cells demonstrate a crucial role of sterol-rich microdomains for Mm-induced damage, cytosolic access, and infection, highlighting the evolutionary conservation of fundamental mechanisms governing mycobacterial virulence and host cell–autonomous immunity.

### Membrane microdomain components are required for EsxA membrane partitioning in vitro

Mm induces membrane damage via ESX-1–mediated secretion of the small peptide EsxA, which interacts with and damages host membranes (*17*, *58*, *59*), possibly with a preference for sterol-rich membranes (*23*, *58*, *60*). To test this hypothesis, we monitored whether recombinant Mtb EsxA partitions into physiological host-derived membranes in vitro (Fig. 8, M and N, and fig. S7, B and C). A whole membrane fraction was purified from wt Dd cells and incubated with rEsxA at different pH values and with different membrane-to-EsxA ratios (fig. S7, B and C). Optimal partitioning was observed at pH 6 (fig. S7B), corresponding to the MCV pH measured inside living Dd using Mm wt coated with fluorescein isothiocyanate (FITC) and tetramethylrhodamine isothiocyanate (TRITC) (fig. S7D). As a control, the pH around an avirulent Mm L1D mutant (*50*) that traffics to phagolysosomes was much lower (fig. S7D). We then incubated rEsxA with membranes extracted from Dd wt and Δ*vacABC* cells treated with or without 10 mM MβCD. We observed a reduction of about 15% of rEsxA association with membranes purified from Dd Δ*vacABC* mutants (Fig. 8, M and N) and a drastic reduction of partitioning into membranes following sterol depletion, down 80% for Dd wt membranes and 90% for membranes from Dd Δ*vacABC* cells. In accordance with the literature, we did not observe association of rEsxB with the membrane fraction under any condition (fig. S7E).

Altogether, these results confirm that the simplest and most plausible consequence of vacuolin absence is a decrease in damage and not a higher Mm restriction, because the MCV in Dd Δ*vacBC*/Δ*vacABC* cells is not more bactericidal, as is it neither more acidic (fig. S8F) nor more degradative (fig. S8, G and H) than in Dd wt cells. Moreover, our results indicate that sterol-rich microdomains are important for EsxA partitioning into host membranes, likely potentiating its membrane-damaging activity.

## DISCUSSION

In Dd, vacuolins are integral membrane proteins that hetero-oligomerize and define specific microdomains of the plasma and phagosomal membranes, similar to their mammalian homologs, flotillins (*38*). Here, we propose that in evolutionarily distant phagocytes, host sterol-rich membrane microdomains are exploited by pathogenic Mm and are required for the efficient membranolytic activity of EsxA, resulting in membrane damage and escape from the MCV.

Vacuolins, particularly VacC, are highly induced both at the mRNA and protein levels during infection with Mm wt. However, their induction is less pronounced and more transient when infected with attenuated mycobacteria such as the Mm ΔRD1 mutant and *M. smegmatis* (Fig. 1). The early induction of *vacC* is likely triggered by common mycobacterial pathogen-associated molecular patterns (PAMPs). However, sustained *vacC* expression requires mycobacteria with functional ESX-1, suggesting a specific response to damage caused by the secretion of membranolytic factors. A similar observation was made in infected murine microglial BV-2 cells using qRT-PCR, showing an up-regulation of Flot-1 at 32 hpi (Fig. 4B). Even though this occurs later than *vacC* in Dd, this indicates that flotillins can also be modulated in response to Mm infection.

Vacuolins are initially present at the MCV in patches, and as the infection progresses, the bacterium becomes surrounded by a densely vacuolin-coated MCV, making catastrophic MCV damage clearly visible. As functional homologs of sterol-binding flotillins, vacuolins are involved in organizing sterol-rich ordered domains. Vacuolins are present in the Triton-insoluble fraction at steady state in uninfected cells, and Mm infection significantly increases this partitioning (Figs. 2 and 3). This biophysical evidence corroborates the transition from a patchy to a solid MCV coat. In addition, sterols also concentrate at the MCV during infection; however, neither GFP-D4H* nor filipin staining show a patchy localization. Whereas vacuolins only associate with microdomains, sterols are core components of membranes even outside of concentrated domains and D4H* and filipin staining, because of their low detection threshold (5% mol/mol for filipin and 20% mol/mol for the D4H), do not differentiate sterol-rich domains from the rest of the membrane, thus explaining the observation of a patchy distribution only for vacuolins. CD treatment leads to a decrease of the filipin staining intensity and to a drastic decrease in vacuolin expression and accumulation at the MCV. Altogether, these findings indicate a specific and interdependent accumulation of the two microdomain components at the MCV, a phenomenon amplified for virulent Mm with full MCV-damaging activity. The use of the LysoFlipper probe in live infected cells allows us to specifically assess biophysical MCV membrane properties in situ. Fluorescence lifetime measurements highlight that the MCV membrane undergoes a profound change in composition with an increase in lipid packing defects compared to endolysosomal compartments, including “standard” latex bead-containing phagosomes probably induced by the increased sterol concentration at the MCV (Fig. 4, C to E). It is important to note that the membrane concentration of sterol can have different impacts on the plasma membrane and endolysosomal membranes. High sterol increases packing of long-chain, unsaturated fatty acids such as those of plasma membrane sphingolipids, but can disorganize endolysosomal membranes containing polyunsaturated fatty acids and lyso-bis-phosphatidic acid. It is also of note that the shedding of some Mm cell wall components such as PDIM, a documented membrane perturbator (*61*, *62*), and their partitioning into the MCV membrane, might also increase packing defects. This is in line with the nonsignificant Δτ measured at the MCV of *M. smegmatis* (Fig. 4D) that does not synthetize PDIM. Therefore, the induction of a strong lipid packing defect due to the combined sterol accumulation, sterol clustering by the presence of vacuolins/flotillins, and the effect of PDIM shedding all increase MCV membrane susceptibility to damaging agents.

Filipin staining is not only observed at the MCV membrane but also enriched inside the MCV in both systems, pointing to a sterol accumulation not only at the limiting membrane but also inside, which might serve as a carbon source for Mm (*57*, *63*, *64*). Under the conditions examined, GFP-D4H* did not label lumenal MCV material, indicating either that the MCVs are not broken or that the damage is not extensive enough to allow the cytosolic probe to access lumenal sterols. Last, MCV proteomic analyses confirm that virulent Mm profoundly affects MCV protein composition compared to nonpathogenic or attenuated mycobacteria. There is, for instance, an important decreased abundance of proteins related to bacteria killing and a higher abundance of proteins linked to lipid acquisition, as well as ESCRT and autophagy machineries (*65*).

We have previously described a role for VacB as a susceptibility factor involved in Mm infection (*8*). However, because the Dd mutant used in that study was an accidental *vacuolin B* and *C* double KO (*38*), here we present a more complete study and dissection of the roles of all three vacuolins and also sterols in the establishment of the Mm replicative niche. We confirm that the absence of VacB, as well as VacA and/or VacC in Dd, and *flotillins* KD in BV-2 confer resistance to Mm infection (Fig. 5). This phenotype was also observed to a similar extent in cells depleted of sterols using CD. We found that the attenuated growth of the Mm ΔRD1 mutant is similar in Dd wt cells, *vacuolin* KO mutants, and sterol-depleted cells. In other words, the absence of vacuolins and sterol depletion specifically affect the growth of Mm wt but not of the Mm ΔRD1 mutant. This corroborates the hypothesis that microdomain components such as sterols and vacuolin/flotillin are specifically exploited by Mm wt and involved in the establishment of the Mm niche in an ESX-1– and membrane damage–dependent manner. The respective impacts of vacuolin absence and CD treatment have different penetrance on the attenuation phenotype, but their effects are additive and most likely do not result from different mechanisms of action. Sterol-rich microdomains spontaneously form in membranes and sterol-binding vacuolins/flotillins likely “only” affect microdomain size and stability. In other words, sterol-rich domains probably exist without organizers, and therefore, sterol depletion/redistribution has the strongest impact on virulence, while the absence of vacuolins/flotillins only decreases it. Altogether, these data indicate that combined perturbations of microdomain components result in an attenuated infection similar to that of the Mm ΔRD1, most plausibly because of a crucial role of membrane microdomains in modulating the escape of Mm from the MCV.

We wondered how sterol-rich microdomains contribute to Mm escape from the MCV. Previous studies showed a decrease in Mtb uptake and growth inside macrophages after CD treatment, presumably by inducing host cell apoptosis and bacteria death (*61*, *66*). In our two infection systems, Dd and BV-2 cells, the addition of CD after Mm uptake allowed us to specifically study the involvement of membrane microdomain components during the intracellular life of Mm. Moreover, under our conditions, we observed neither increased host cell death after treatment with MβCD nor precocious bacteria release. By performing RNA-seq analyses of Dd, we further confirmed that CD treatment does not induce significant changes in the transcriptional profile of mock-infected cells (Fig. 6). Moreover, RNA-seq analysis also clearly indicates that absence of vacuolins and sterol depletion dampen the up-regulation of ESCRT- and autophagy-related genes in response to infection. This dampening of the transcriptional response is also clearly visible when analyzing genes encoding ubiquitin E3 ligases of the TRAF family, among which TrafE is known to act upstream of the ESCRT and autophagy and coordinate their action in repair. The TrafE transcript is clearly up-regulated during infection, but CD treatment strongly dampens this response as early as 6 hpi, inducing a clustering of this condition with the mock-infected samples (https://zenodo.org/records/15111828). Altogether, the simplest and most plausible hypothesis is that altering sterol-rich vacuolin/flotillin microdomains directly prevents Mm-induced MCV damage.

We and others use LLOMe as a proxy to understand how mycobacteria-induced MCV damage is sensed and repaired by the host cell (*21*, *53*, *54*). Because activation of LLOMe requires an acidic and proteolytic environment, we monitored that CD treatment does not significantly alter these properties in both Dd and BV-2 cells. Using LysoSensor to follow endolysosomal leakage and two markers of damage sensing and repair, Alix and the ESCRT-III subunit Vps32 in Dd (*20*, *21*) and Gal3 in BV-2 cells, we show that the presence of microdomain components is required for LLOMe to induce endolysosomal damage. This interpretation is further strengthened by monitoring cell death as a final consequence of endolysosomal damage. Perturbations of membrane microdomain components increase cell survival to LLOMe-induced damage. Notably, CD treatment of Dd Δatg1Δtsg101 cells, which are defective in ESCRT and autophagy-mediated repair, increases cell viability, which can only be explained by a reduction in damage and not enhanced repair. Together, these results demonstrate that membrane composition affects the action of this lysosomotropic membrane disrupter (Fig. 7). However, it is important to note that LLOMe-induced damage may not fully recapitulate the specific and complex molecular interactions of EsxA with the MCV.

**Fig. 9.**
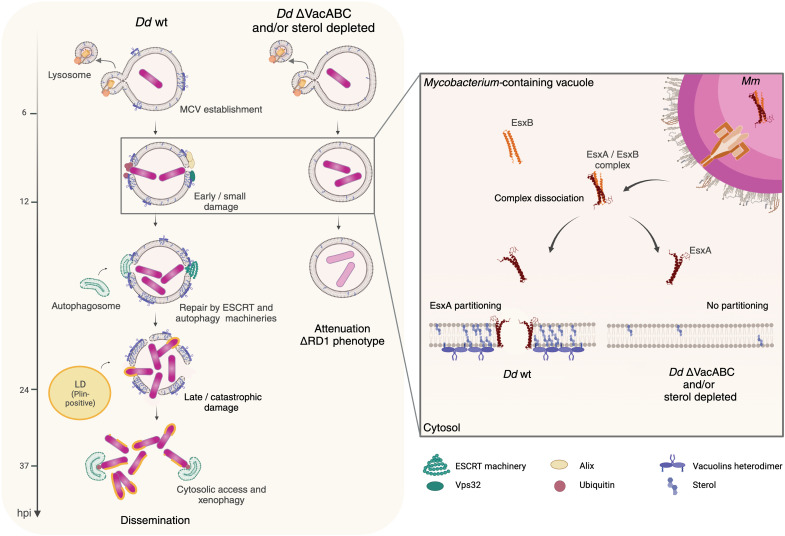
Working model of the role of membrane microdomains during *M. marium* infection. Briefly, sterol-rich membrane microdomains accumulate at the MCV during infection, facilitating EsxA partitioning into the MCV membrane, and subsequent damage and Mm escape to the cytosol. Disruption of microdomains inhibits partitioning of EsxA into the MCV membrane, thereby limiting damage and leading to an attenuated infection. Generated with BioRender (https://BioRender.com/mdmfsjt).

Membrane microdomain components also play a role in facilitating Mm-induced MCV damage. We showed that the levels of ubiquitination in the vicinity of the bacterium, Vps32 (in Dd) and Gal3 (in BV-2) recruitment, which are among the earliest signs of damage and repair, are lower under CD treatment, suggesting that sterols are important for the induction of early and/or small damage in both host models (Fig. 8). In addition, Plin binding to Mm, a hallmark of extensive catastrophic damage and cytosolic access, is strongly affected in sterol-depleted cells and appears to be further reduced in Dd *vacuolin* KO cells. Altogether, our evidence indicates two distinct stages in MCV damage progression and that interference with microdomains reduces damage and cytosol escape, leading to a confinement that is sufficient to strongly attenuate Mm wt intracellular growth in both Dd and BV-2 cells (see the model in Fig. 9). Note that the observations of such infection phenotypes and biological processes in murine microglial cells are likely relevant for research on extra-pulmonary TB. The devastating tuberculous meningitis is the most severe form of tuberculosis with a fatality rate of 20 to 50% in treated individuals (*67*).

Mm and Mtb secrete EsxA, which has long been described as a membrane-disrupting toxin (*17*, *58*, *68*, *69*). EsxA is secreted as a dimer with EsxB, its putative chaperone, through the ESX-1 secretion system. To damage the membrane, EsxA must first dissociate from EsxB, which occurs at low pH and was recently shown to require acetylation of EsxA (*58*, *70*). During Mm infection of Dd, the average MCV pH remains around 6 (fig. S8D), probably due to early membrane damage leading to proton leakage. In addition, we previously documented that some Mm experience a transient drop in pH, because a fraction of MCVs are LysoSensor positive at an early stage of infection (*19*), which stimulates the map24::GFP transcriptional reporter (*8*). We assume that a slightly acidic pH allows the dissociation of Mm EsxA from its chaperone. Moreover, in vitro partitioning of EsxA in the MCV membrane strongly depends on the presence of vacuolins and the level of sterols (Fig. 8, M and N). We propose that the progression of damage observed at the MCV mirrors the accumulation of vacuolins/flotillins and sterols that are likely responsible to create a membrane environment riddled with lipid packing defects that favor the activity of membrane damaging agents. Interference with membrane microdomain components phenocopies the attenuation of the Mm ΔRD1 mutant. Therefore, vacuolins and sterols behave as susceptibility factors, and their expression/accumulation is induced by Mm-triggered damage, which creates an amplification loop that is fully interrupted in the combined absence of vacuolins and cyclodextrin treatment. Other additional virulence factors, such as PDIMs, reported to induce sterol clustering (*23*–*25*, *71*), likely also contribute to create the optimal membrane environment for EsxA activity.

In this study, we showed that the involvement of membrane microdomains during Mm infection extends beyond the Dd infection model and is also observed in mammalian phagocytes. Consistently with our findings, recent work has revealed flotillins as a major determinant of the fate of Mtb infection in patients, because overexpression of human flotillin-1, resulting from particular allele variants, acts as a host susceptibility factor for Mtb infection (*72*). Last, we propose that Mm actively modulates the composition and properties of the MCV membrane by manipulating host membrane microdomain proteins and lipids. This, in turn, enhances EsxA partitioning and membranolytic activity, allowing Mm escape from the MCV and thereby promoting growth and dissemination in distantly evolutionarily related phagocytes, such as the amoeba Dd and murine microglial cells (Fig. 9).

## MATERIALS AND METHODS

### Dd culture

Dd strains and plasmids are listed in table S1. Cells were axenically grown at 22°C in HL5c medium (Formedium) supplemented with penicillin (100 U/ml) and streptomycin (100 μg/ml; Invitrogen). Plasmids were transfected into Dd by electroporation and selected with the relevant antibiotic. Hygromycin was used at a concentration of 15 μg/ml for KO cell lines and 50 μg/ml for cell lines with reporters integrated at the safe-haven act5 locus. Blasticidin was used at a concentration of 5 μg/ml for KI and KO cell lines.

### BV-2 cell line culture

BV-2 cell lines and vectors are listed in table S1. Cells were grown at 37°C in Dulbecco’s Modified Eagle Medium (DMEM) complemented with 10% fetal bovine serum (FBS), penicillin (100 U/ml), and streptomycin (100 μg/ml; Invitrogen). Stable KD cell lines were produced by lentiviral transduction of vectors containing the sequence of short hairpin RNA targeting the genes of interest and selected with puromycin at a concentration of 10 μg/ml.

### Mycobacterial culture conditions

The mycobacterial strains used in this study are listed in table S1. Mycobacteria were grown in Middlebrook 7H9 (Difco) supplemented with 10% Oleic Albumin Dextrose Catalase (OADC)(Becton Dickinson), 0.2% glycerol, and 0.05% Tween 80 or tyloxapol (Sigma Aldrich) at 32°C in shaking culture at 150 rpm in the presence of 5-mm glass beads to prevent clumping. Hygromycin was used at a concentration of 100 μg/ml for mCherry/GFP expression, and kanamycin was used at a concentration of 50 μg/ml for GFP/DsRed expression or 25 μg/ml for lux expression. To follow Mm in vitro growth, 2 × 10^5^ bacteria were plated in 200 μl of 7H9 with 10% OADC, 0.05% tyloxapol, and 0.2% glycerol in a 96-well plate. The GFP signal was measured with a Synergy Mx Monochromator-Based Multi-Mode Microplate Reader (Biotek) at 32°C with constant orbital shaking in a black 96-well plate (PerkinElmer).

### Antibodies and reagents

Recombinant nanobodies with the Fc portion of rabbit immunoglobulin G (IgG), which specifically recognize VacA or VacB, were previously characterized (*38*). Other antibodies used included pan-vacuolin 221.1.1 [M. Maniak (*36*)], ubiquitin FK2 (Enzo Life Sciences), GFP (pAb from MBL Intl. and mAb from Abmart), cathepsin D [J. Garin (*73*)], adipophilin (Plin; Biosynth), and Filipin III from *Streptomyces filipinensis* (Sigma-Aldrich). Goat anti-mouse or anti-rabbit IgG coupled to AlexaFluor 488, AlexaFluor 594, AlexaFluor 647 (Invitrogen), or horseradish peroxidase (HRP) (Brunschwig) was used as secondary antibodies. To stain acidic compartments, 1 μM LysoSensor Green DND-189 (Thermo Fisher Scientific), a pH-dependent probe that becomes more fluorescent in acidic compartments, was added to the infected cells. After 10 min of incubation, excess dye was washed off depending on the assay and cells were imaged for a maximum of 30 min. In BV-2, BV-2 LTR was used at 50 nM and incubated for 3 hours before imaging. To stain compartments with proteolytic activity, DQ Green BSA (50 μg/ml; Thermo Fisher Scientific), which releases fluorescent protein fragments upon proteolysis of the self-quenched BSA-associated Bodipy dye, was added to the infection sample 1 hour before imaging.

### Western blotting

After SDS–polyacrylamide gel electrophoresis separation and transfer onto nitrocellulose membranes (Protran), immunodetection was performed as previously described (*74*) but with ECL Prime Blocking Reagent (Amersham Biosciences) instead of nonfat dry milk. Detection was performed with ECL Plus (Amersham Biosciences) using a Fusion Fx device (Vilber Lourmat). Quantification of band intensity was performed with ImageJ.

### Immunofluorescence

For immunofluorescence, infected Dd cells were fixed with ultracold methanol at the indicated time points and immunostained as previously described (*75*). BV-2 cells were fixed with 4% paraformaldehyde (PFA) in Phosphate-Buffered Saline (PBS) for 20 min and then washed three times in PBS before antibody staining. Images were recorded with a Leica SP8 confocal microscope using 63 × 1.4 numerical aperture (NA) oil immersion objectives or Leica Stellaris 8 63 × 1.4 NA oil immersion objectives or with a 40× water immersion objective with the ImageXpress Micro XL HC microscope. For filipin staining, cells were fixed in a solution of 4% PFA/picric acid as previously described (*75*). Then, cells were stained in a solution of filipin (50 μg/ml) in PBS in the dark for 30 min at room temperature. Images were recorded with a spinning disc confocal system (Intelligent Imaging Innovations) mounted on an inverted microscope (Leica DMIRE2; Leica) using the 100 × 1.4 NA oil objective or Leica Stellaris 8 63 × 1.4 NA oil immersion objectives.

### LysoFlipper assay

After infection, BV-2 cells were incubated with 2 μM LysoFlipper in FluoroBrite medium for 1 hour before imaging. Imaging was performed at the Leica SP8 DIVE Falcon upright microscope with 25 × 0.95 NA water immersion objective. FLIM imaging was performed at λ = 550 to 700 nm with two line accumulations and three frame repetitions. Images were analyzed using LasX software and the fluorescence decay was fitted to a biexponential equation. The presented data represent the longer lifetime (τ_2_) of the whole cell compared to the region of interest (ROI = MCV or bead phagosome).

### Dd infection assay

Infections were performed as previously described (*8*, *76*) with few modifications. To compensate for phagocytosis defect, Δ*vacABC* cells were infected with 1.5× more bacteria than Dd wt cells. After infection and phagocytosis, extracellular bacteria were washed off, and attached infected cells were resuspended in filtered HL5c containing a bacteriostatic dose of streptomycin (5 μg/ml) and penicillin (5 U/ml) to prevent growth of extracellular Mm. Mock-infected cells were treated similarly but without bacteria. The indicated concentration of MβCD (Sigma-Aldrich) was added after bacteria phagocytosis to prevent any effect on Mm uptake. The intracellular growth was monitored by measuring the luminescence signal with a Synergy Mx Monochromator-Based Multi-Mode Microplate Reader (Biotek) at 25°C in a white 96-well plate. For quantification of the damage/repair reporters, cell imaging was performed with a 60× water immersion objective with the ImageXpress Micro XL HC microscope at the indicated time points using three wells per condition with nine imaged fields per well with three z-sections per field with a 1-μm interval. Analysis was performed per cell after segmentation with the MetaExpress software as previously described (*77*).

### BV-2 infection assay

The same protocol was followed for BV-2 infections, with modifications. The day before the infection, 5 × 10^5^ cells/ml were plated in DMEM complemented with FBS. Cells were infected at a multiplicity of infection of 5 and maintained at 32°C. For CD experiments, before infection, the medium was changed to DMEM complemented with delipidated FBS (LRA Sigma no. 13358-U) and the indicated concentration of MβCD or HβCD (Sigma-Aldrich) was added after phagocytosis. Infection was monitored by microscopy with a 40× objective with the ImageXpress Micro XL HC microscope every 1.5-hour interval for 48 hours using three to four wells per condition with nine imaged fields per well with three sections per field with a 1-μm interval. Analysis was performed with the MetaExpress software and quantifying area of the GFP signal.

### Phagocytic plaque assay

Dd plaque formation on a lawn of *Klebsiella pneumoniae* (GE) mixed with mycobacteria was monitored as previously described (*78*). A 5 × 10^8^ mycobacteria/ml culture was centrifuged and resuspended in 1.2 ml of 7H9 containing a 1:10^5^ dilution of *K. pneumoniae* grown overnight in LB. Fifty microliters of this suspension was deposited on wells from a 24-well plate containing 2 ml of 7H10-agar (without OADC). Serial dilutions of Dd (10, 10^2^, 10^3^, or 10^4^ cells) were plated onto the bacterial lawns, and plaque formation was monitored after 4 to 7 days at 25°C.

### Quantitative real-time polymerase chain reaction

RNA from mock-infected cells or cells infected with Mm wt, ΔRD1, or *M. smegmatis* was extracted at the indicated time points using the Direct-zol RNA MiniPrep kit (Zymo Research) following the manufacturer’s instructions. RNA (1 μg) was retrotranscribed using the iScript cDNA Synthesis Kit and polydT primers (Bio-Rad).

The cDNA was amplified using the primers listed in table S2 and the SsoAdvanced universal SYBR Green supermix (Bio-Rad). Amplimers for vacA, vacB, vacC, and gapdh were detected on a CFX Connect Real-Time PCR Detection System (Bio-Rad). The housekeeping gene gapdh was used for normalization. PCR amplification was followed by a DNA melting curve analysis to confirm the presence of a single amplicon. Relative mRNA levels (2^−ΔΔCt^) were determined by comparing first the PCR cycle thresholds (Ct) for the gene of interest and gapdh (ΔC) and, second, Ct values in infected cells versus mock-infected cells (ΔΔC).

Following infection with GFP-expressing bacteria, the cell population was pelleted and resuspended in 500 μl of HL5c, passed through 30-μm filters, and sorted by FACS (Beckman Coulter MoFlo Astrios). The gating was based on cell diameter (forward scatter) and granularity (side scatter). Infected (GFP-positive) sub-fraction was selected based on the GFP intensity (FITC channel). Mock-infected cells were subjected to the same treatments and collected as GFP negative. Typically, ~5 × 10^5^ cells of each fraction were collected for RNA isolation. RNA isolation was performed as for qRT-PCR. Quality of RNA libraries, sequencing, and bioinformatic analysis were performed as previously described (*39*).

### Induction of sterile damage with LLOMe

For LLOMe experiments, 5 × 10^5^ cells were plated in 96-well IBIDI dishes in filtered HL5c. To stain acidic compartments, 1 μM LysoSensor Green DND-189 (Thermo Fisher Scientific) was added to the cells. After 45 min of incubation, excess dye was washed away. Cells were imaged with a spinning disc confocal system (Intelligent Imaging Innovations) mounted on an inverted microscope (Leica DMIRE2; Leica) using the 100 × 1.4 NA oil objective every minute for 1.5 hours. For quantification of the repair reporters, cell imaging was performed with a 60× water immersion objective with the ImageXpress Micro XL HC microscope every 5 min for 1.5 hours using three wells per condition with four image fields per well with three sections per field with a 1-μm interval in three biological replicates of the experiment. Briefly, a first image was taken for all conditions before LLOMe addition at the indicated concentration, followed by the localization of GFP-Vps32 and Alix-GFP. For MβCD treatment, cells were incubated with 2 mM MβCD 40 min before LLOMe addition. Analysis was performed per cell and dot formation and area were quantified with the MetaExpress software as previously described (*77*). Similarly, BV-2 experiment was performed by plating 2.5 × 10^5^ cells the day before the experiment in DMEM containing delipidated FBS, and CD was added 1 hour before the assay at the indicated concentrations. Images were acquired with a 40× water immersion objective with the ImageXpress Micro XL HC microscope every 20 min for 3 hours using three wells per condition with four image fields per well with three sections per field with a 1.5-μm interval in three biological replicates of the experiment.

### Cytosol-membrane separation and rEsxA incubation

Dd cells (10^9^) were washed in Sorensen-Sorbitol and resuspended in HESES buffer (20 mM Hepes, 250 mM sucrose, 5 mM MgCl^2^, and 5 mM ATP) supplemented with protease inhibitors (cOmplete EDTA-free, Roche). Cells were homogenized using a ball homogenizer with a 10-μm clearance. The postnuclear supernatant was then diluted in HESES buffer and centrifuged at 35,000 rpm in a Sw60 Ti rotor (Beckman) for 1 hour at 4°C. The cytosol (SN, supernatant) and membrane (MB, pellet) fractions were subsequently collected.

The protein concentration of the cytosol fraction was quantified using the Bradford method. Different quantities of membranes were tested as detailed in fig. S6. Ultimately, 400 μg of membranes was incubated with 12 μg of recombinant EsxA (rESAT-6, BEI Resources NR-49424) in HESES buffer at pH 6 for 20 min at room temperature on a wheel. After ultracentrifugation at 45,000 rpm for 1 hour at 4°C in a TLS-55 rotor (Beckman), the membranes were separated into SN and pellet (P) fractions. Equal amounts of SN and P were loaded for Western blotting.

### Detergent-resistant membrane isolation

Dd cells (10^8^) were washed in Sorensen-Sorbitol and resuspended in 1 ml of cold lysis buffer (50 mM tris-HCl, pH 7.5, 150 mM NaCl, 50 mM sucrose, 5 mM EDTA, 5 mM ATP, and 1 mM DTT) with 1% Triton X-100 supplemented with protease inhibitors. The lysate was incubated at 4°C on a rotating wheel for 30 min. After centrifugation at 13,000 rpm for 5 min in a tabletop centrifuge at 4°C, the supernatant (TSF) was collected, and the pellet (TIF) was resuspended in 200 μl of cold lysis buffer without Triton X-100.

The TIF was mixed with 800 μl of 80% sucrose (to achieve a final concentration of 65% sucrose) and deposited at the bottom of an ultracentrifuge tube. It was then overlaid with 2 ml of 50% sucrose and 1 ml of 10% sucrose. The sample was centrifuged at 55,000 rpm in an Sw60 Ti rotor (Beckman) for 2 hours at 4°C. The TIFF was collected, acetone precipitated, and resuspended in Laemmli buffer. Equivalent amounts of each fraction (TSF, TIF, and TIFF) were loaded for Western blotting.

### Image analysis and statistical analysis

To quantify the percentage of cells with high vacuolin signal, we segmented cells based on the bright-field image. Infected cells were identified based on the presence of intracellular bacteria. Then, the GFP intensity of infected and bystander cells was extracted as intensity average per cell. An arbitrary threshold of intensity (<5000), corresponding to the maximum intensity measured in mock cells, was chosen to filter for low or high expressing cells. To quantify filipin intensity at the MCV, we segmented intracellular bacteria based on the presence of a bacteria mask in a cell mask. Then, the intracellular bacteria mask was grown by one pixel and filipin intensity was measured in the grown bacteria mask. To quantify the percentage of infected cells positive for the different reporters in Dd, Mm was segmented based on its fluorescent tag (mCherry or GFP) and cells were segmented on the bright-field or fluorescence channel depending on the expression level. Then, the recruitment of reporters was detected first by identifying structures with signal intensity higher than the cytosol and subsequently colocalization of these structures with the Mm mask (pixel overlap with the bacteria). For BV-2 quantification, Mm was segmented based on its fluorescent tag (mCherry or GFP) and cells were segmented using the bright-field and fluorescence channels. Then, we identified intracellular bacteria based on their localization in the cell mask. The intracellular bacteria masks were grown by 1 pixel, and the recruitment of reporters was detected first by identifying structures with signal intensity higher than the cytosol and subsequently colocalization of these structures with the Mm mask (pixel overlap with the bacteria). Plin/bacteria colocalization used a similar segmentation procedure, but we analyzed the percentage of surface overlap between the Plin mask area and the grown bacteria mask area. A similar segmentation was performed to identify formation of dots after LLOMe treatment in Dd and BV-2 (without bacteria mask). The BV-2 cells could not be segmented as single cells; therefore, the Gal3 dots area was normalized to the total cell mask area. To measure Mm growth in BV-2 cells, we segmented Mm as previously carried out and quantified the total bacterial area.

The sample size and *P* values are presented in appropriate figure legends (*n* = number of technical replicates/*N* = number of biological replicates). All statistical analyses were performed with the GraphPad Prism 10 software and plots present the mean ± SEM. The level of significance is indicated as *P < 0.05; **P < 0.01; ***P < 0.005, ****P < 0.0001 and “ns” indicates no significant difference.

## Supplementary Materials

### The PDF file includes:

Figs. S1 to S8 Tables S1 and S2 Legends for movies S1 and S2 References
