# Supplementary material for: Membrane microdomains are crucial for Mycobacterium marinum EsxA-dependent membrane damage, escape to the cytosol, and infection
Source: Sci Adv. 2026 Jan 1;12(1):eady0812. doi: 10.1126/sciadv.ady0812 (PMC12757022; doi:10.1126/sciadv.ady0812)
Supplement: Supplementary file 1 — Figs. S1 to S8 Tables S1 and S2 Legends for movies S1 and S2 References [file sciadv.ady0812_sm.pdf]

Science Advances
AAAS

Supplementary Materials for

Membrane microdomains are crucial for *Mycobacterium marinum* EsxA-dependent membrane damage, escape to the cytosol, and infection

Angélique Perret *et al.*

Corresponding author: Thierry Soldati, thierry.soldati@unige.ch

Sci. Adv. **12**, eady0812 (2026)
DOI: 10.1126/sciadv.ady0812

The PDF file includes:

Figs. S1 to S8
Tables S1 and S2
Legends for movies S1 and S2
References

Other Supplementary Material for this manuscript includes the following:

Movies S1 and S2

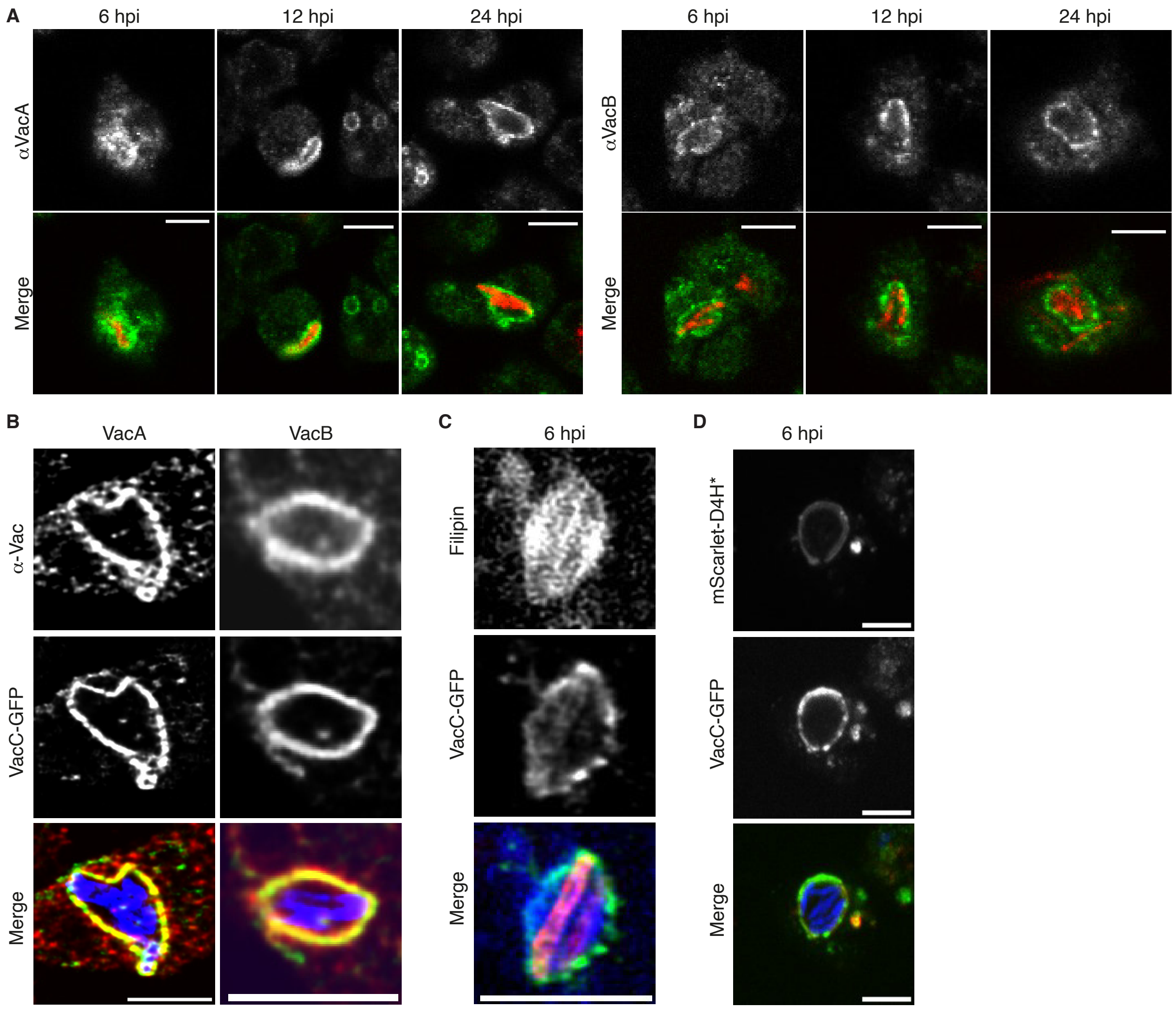

Figure S1: Endogenous VacA, VacB and sterols are present at the MCV.

A. Representative images of wt cells infected with GFP-expressing Mm wt, fixed at indicated time points and immunostained with recombinants antibodies against endogenous VacA or VacB. Scale bar, 5 µm. **B.** Representative images of VacC-GFP KI cells infected with BFP-expressing Mm wt, fixed and immunostained with recombinants antibodies against endogenous VacA or VacB. Scale bar, 5 µm. **C.** Representative image of wt cell expressing GFP-D4H* infected with mCherry-expressing Mm wt, fixed and stained with Filipin. Scale bar, 5 µm. **D.** Representative live images of VacC-GFP KI expressing mScarlet-D4H* infected with BFP-expressing Mm wt at 6 hpi. Scale bar, 5 µm.

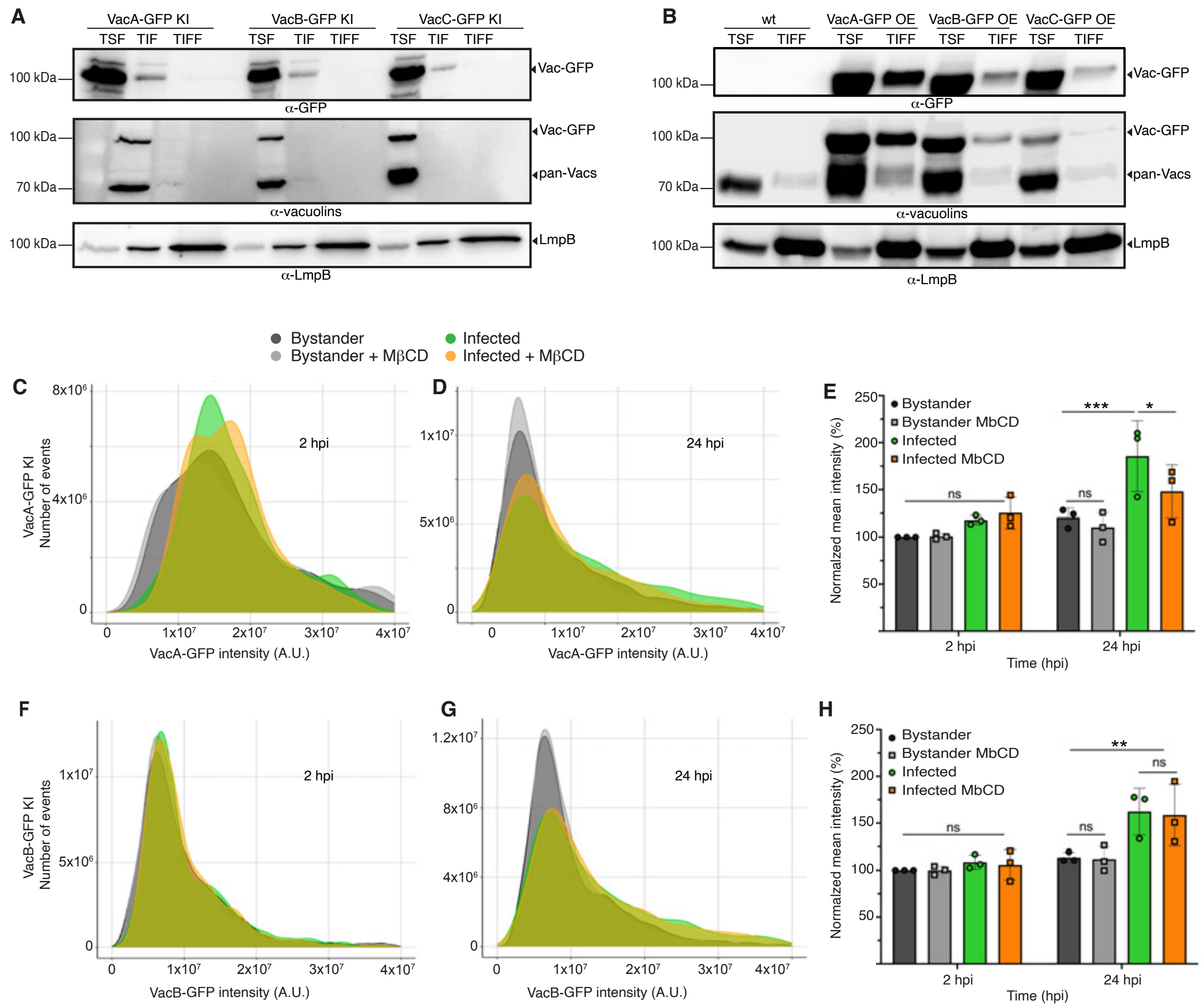

Figure S2: Membrane microdomain components accumulate at the MCV during infection.

A-B. Indicated non-infected Vac-GFP KI (**A.**) or Vac-OE (**B.**) cells were lysed in cold Triton X-100. The Triton soluble (TSF) and insoluble (TIF) fractions were recovered, as well as the floating fraction (TIFF) from a sucrose gradient. **C-H.** Indicated Vac-GFP KI cells were infected with expressing-mCherry Mm wt and GFP intensity was measured at the indicated time points. **C and H.** Normalized distribution of the indicated Vac-GFP KI intensity during infection and treatment with 2 mM of MβCD at 2 hpi (**C**. and **F.**) and 24 hpi (**D**. and **G.**) (N=3, n≥ 50000 cells). **E.** and **H.** Mean intensity of C-D. and F-G. (mean + sd, N=3, two-way ANOVA - Tukey test, *p≤0.05, **p≤0.01, ***p≤0.005).

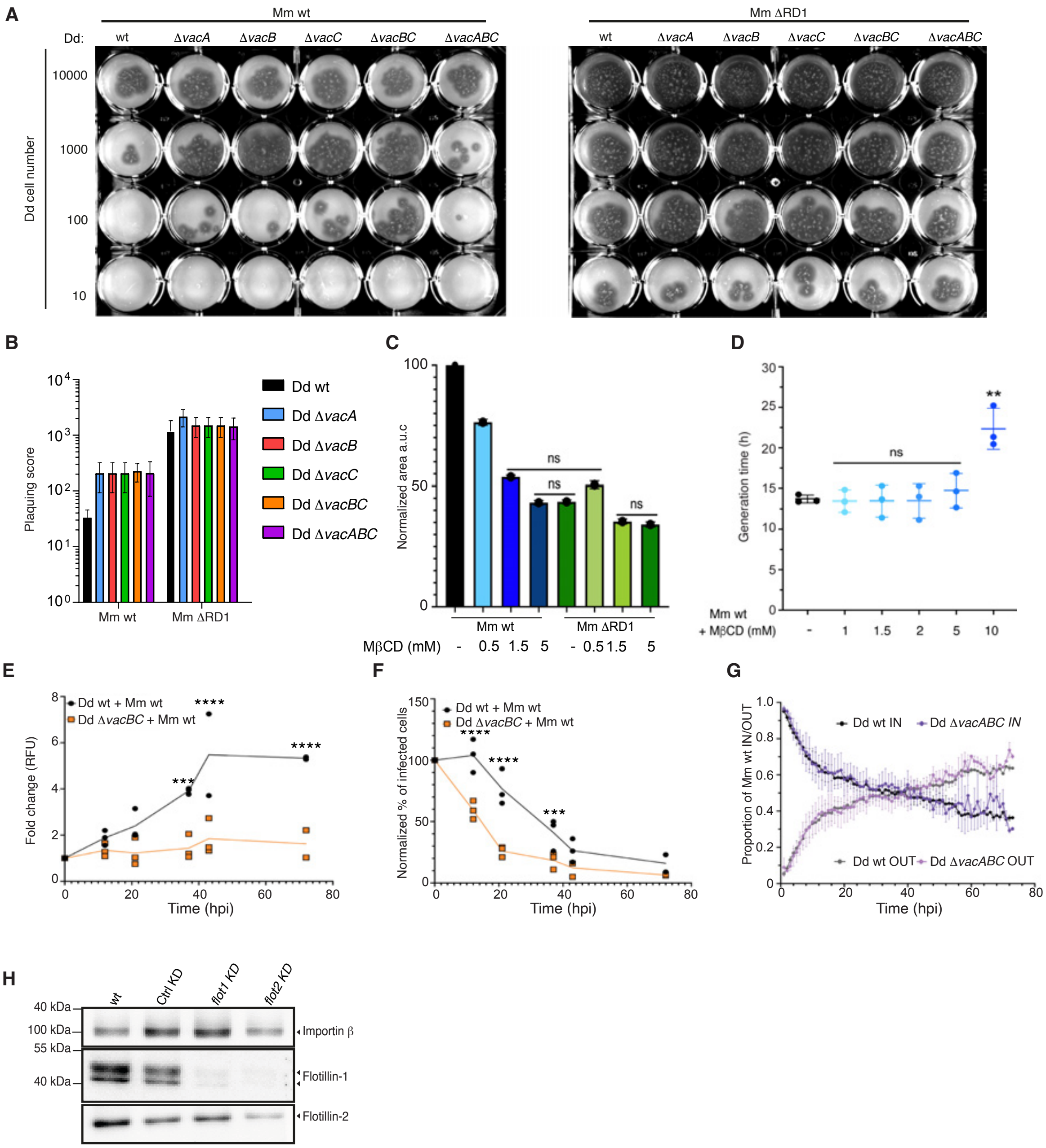

Figure S3: Plaque assays with vacuolin KO cells and dose-dependent effect of MβCD.

A-B. Dilutions of wt or *vacuolin* KO cells were deposited on the lawns of *K. pneumoniae* mixed with indicated mycobacterial. **A.** Representative images of plaque assays taken at day 5. **B.** The plaquing score was determined using a logarithmic scale. (mean ± sem, N=4). **C.** Wt cells were infected with bioluminescent Mm wt or ΔRD1 and treated with increasing doses of MβCD and luminescence was monitored for 72 hours (mean of the area under the curve ± sd N=3, one-way ANOVA- Dunnett test, *p≤0.05, **p≤0.01, ***p≤0.005, ****p≤0.0001). **D.** Generation time of GFP-expressing Mm wt monitored for 72 hours in a plate reader (mean fold change ± sd N=3, one-way ANOVA - Dunnett test, **p≤0.05). **E-F.** Wt and Δ*vacBC* cells were infected with GFP-expressing Mm up to 72 hours. At the indicated time

points, fluorescence of infected cells (**E.**) and percentage of infected cells (**F.**) were measured by flow cytometry (mean ± sd, N=3, two-way ANOVA - Fisher's LSD test, *$p \leq 0.05$, **$p \leq 0.01$, ***$p \leq 0.005$, ****$p \leq 0.0001$). **G.** Wt and Δ*vacABC* cells were infected with GFP-expressing Mm wt and imaged by HC microscopy for 72 hours. After segmentation, the proportion of intracellular and extracellular bacteria calculated for each time point (mean fold change ± sem, N=3). **H.** Total protein lysates of BV-2 cells, transduced with vectors expressing shRNAs to knock down Flot-1 or Flot2, were subjected to western blot assays using antibodies against Flot-1 or Flot-2. As loading control, samples were probed with anti-Importin-β.

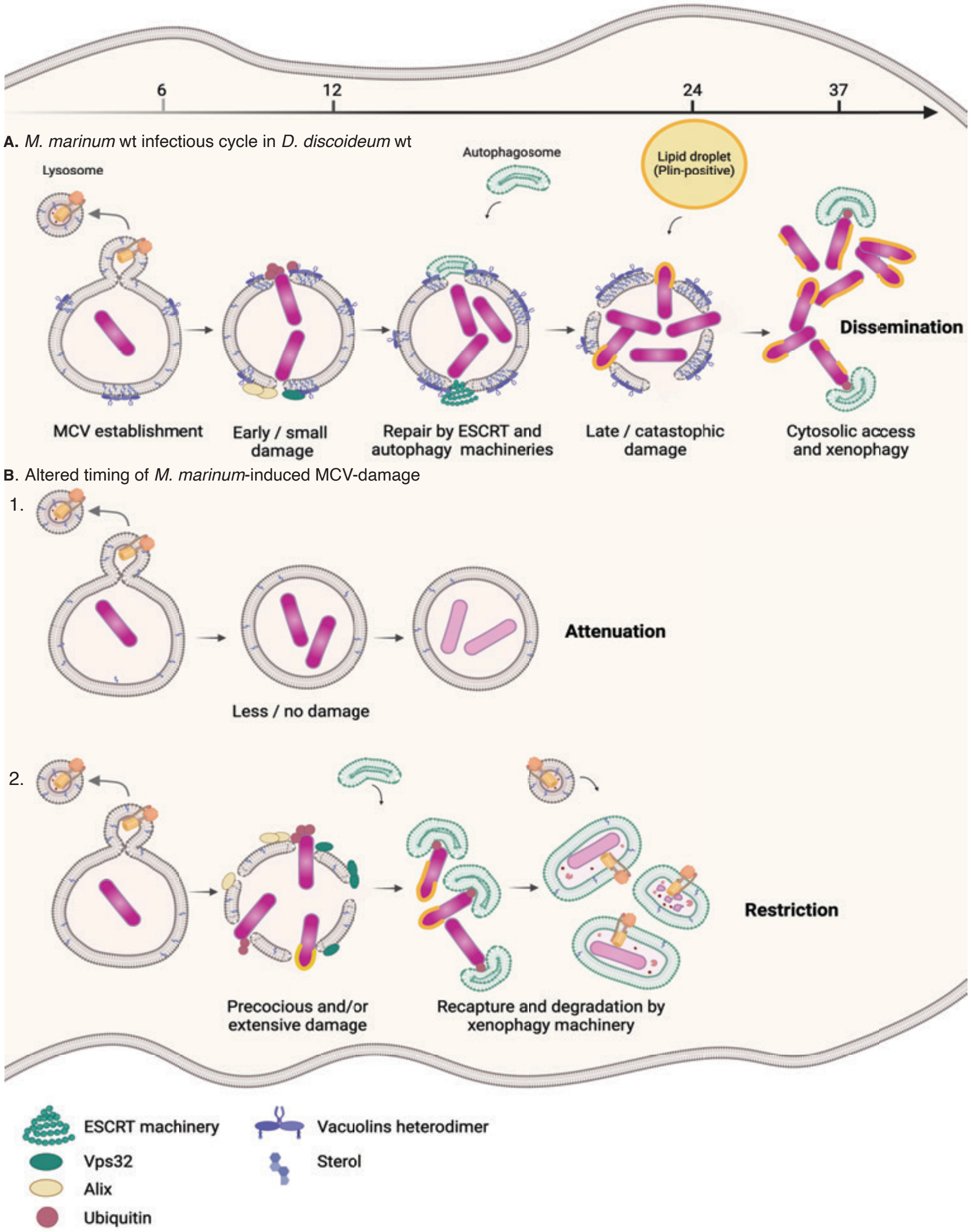

Figure S4: Model depicting different possible scenario for increased resistance to infection.
A. Infection course of Mm wt in wt cells. After entry by phagocytosis, Mm blocks phagosome maturation and establishes an MCV. At early stages, Mm induces damage to the MCV membrane, damage is sensed and repaired by the ESCRT and autophagy machineries. When catastrophic level of damage is reached, Mm escapes to the cytosol where perilipin (Plin) binds their now accessible hydrophobic surface. Mm continues to replicate before it egresses and infects neighbouring cells. **B.** Attenuation can be a consequence of no (or less) MCV damage leading to Mm containment in the MCV or phagosome, possibly inhibiting growth by limiting access to nutrients (**B.1.**). Alternatively, attenuation can also result from more efficient damage leading to precocious escape to the cytosol followed by recapture and restriction by the xenophagy machinery (**B.2.**). Generated with BioRender.

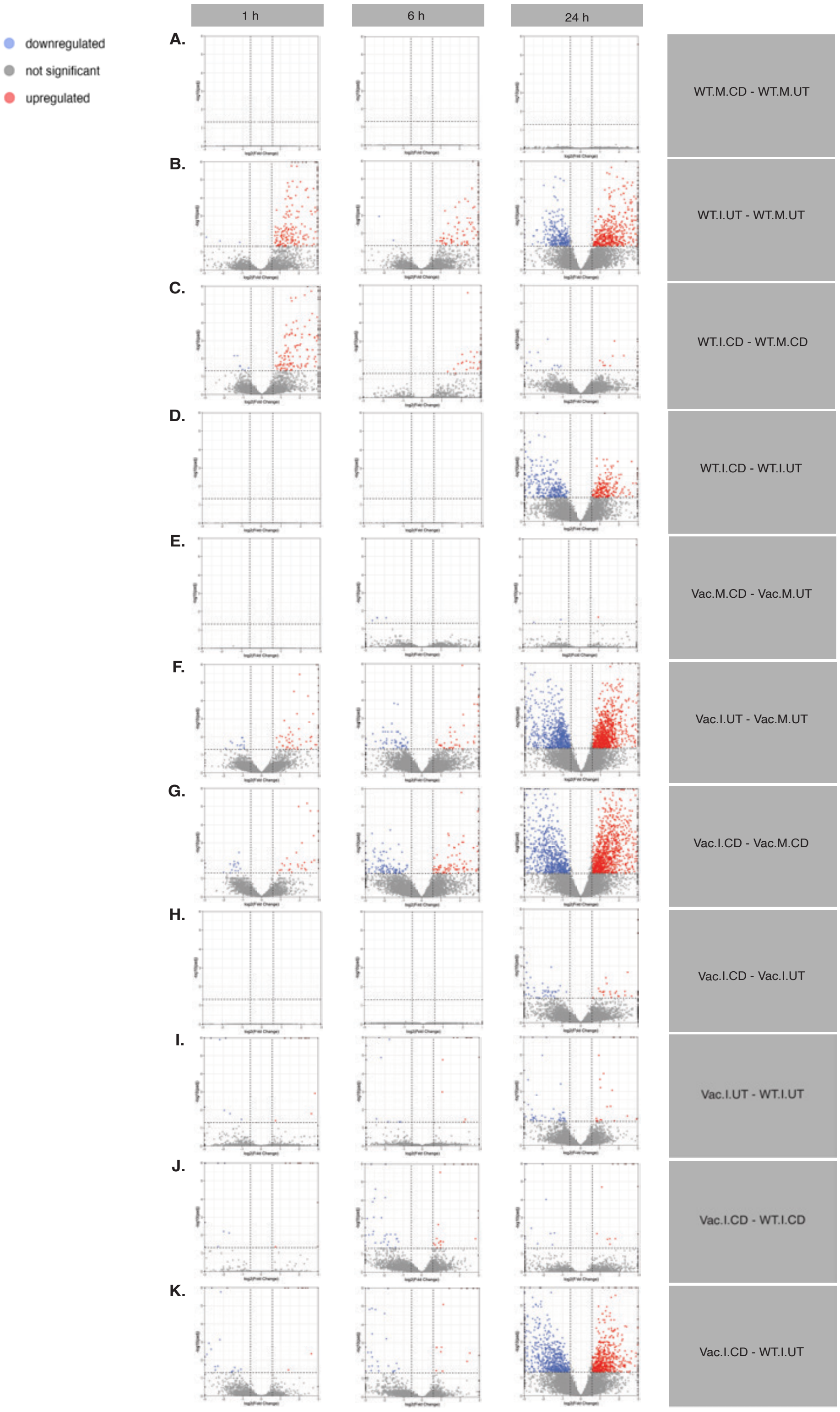

Figure S5: Volcano plots of all RNAseq analyses, ordered by conditions and time points. Selected cut-off for statistical significance is abs(log2FC)>0.5848 and padj <0.05. Blue dots represent downregulated genes, and red dots represent upregulated genes. WT: Dd wt, Vac: Dd Δ*vacABC*, M: Mock, I: Infected, UT: Untreated, CD: MβCD, 1,6 and 24h: Time point post infection (hpi).

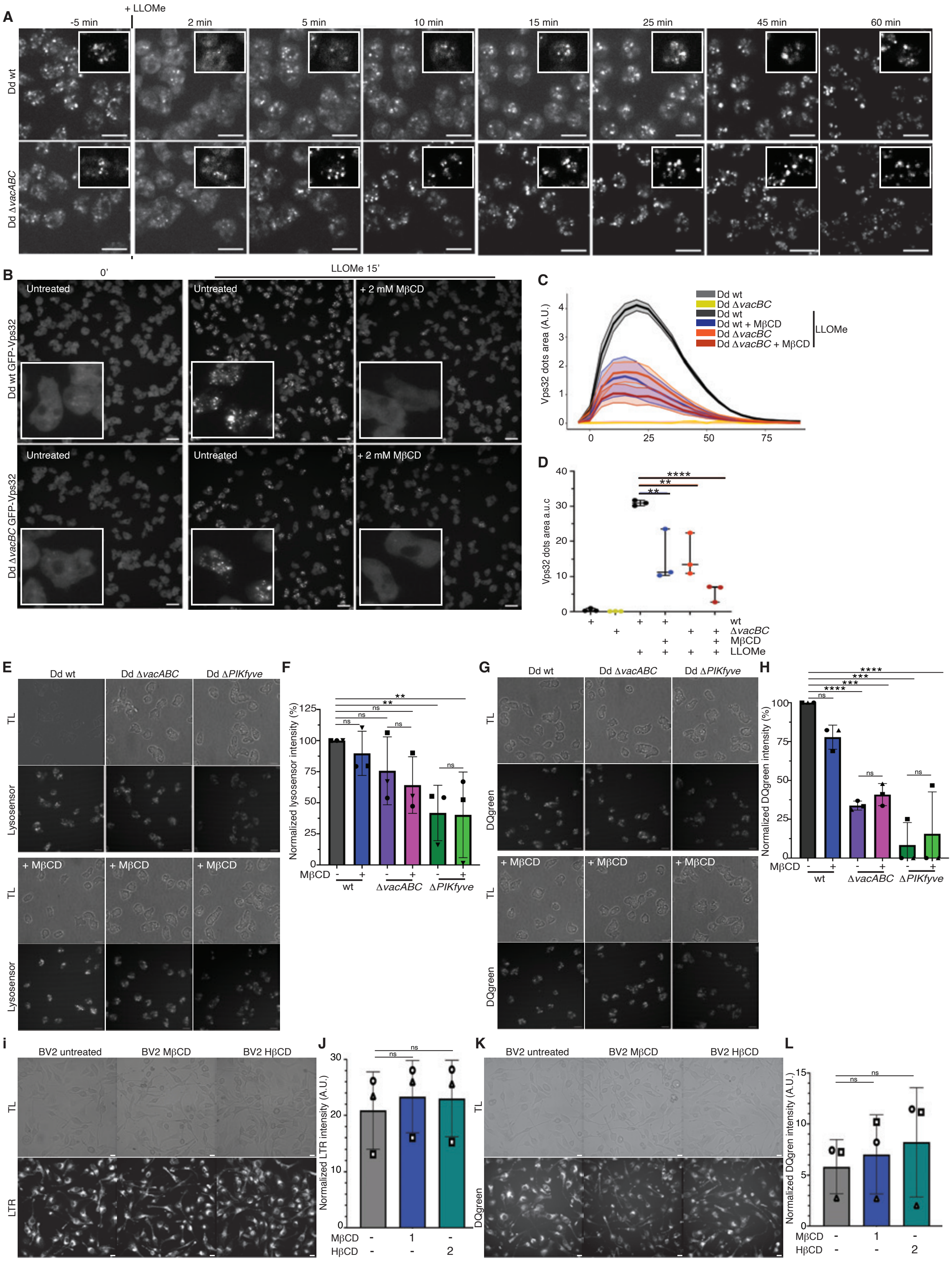

Figure S6: Vacuolin KO cells react less and recover faster from LLOMe treatment.

A. Time-lapse images of movie S1 and S2 of wt and Δ*vacABC* cells stain with lysosensor and treated with 4.5 mM of LLOMe for a total of 90 min. Insets are magnified 2-fold. Scale bar, 5 µm. **B-D.** Dd wt and Δ*vacBC* cells expressing GFP-Vps32, treated or not with 2 mM of MβCD, were submitted to 4.5 mM of LLOMe. **B.** Representative live images of cells before treatment and 25 min after LLOMe addition, insets are magnified 3.5-fold Scale bar, 5 µm. **C.** One representative experiment showing the area of Vps32 dots through time. The analysis is running per cell with <600 cells per condition. **D.** Area under the curve of C. (mean ± s.d, n=3; N=3, One-way ANOVA - Dunnett test, ***p≤0.005, ****p≤0.0001). **E-H.** Dd wt, Δ*vacABC* and ΔPIKfyve untreated of treated with 2mM of MβCD were incubated with lysosensor or DQgreen-BSA for 3h and imaged live by HC microscopy. **E.** Representative images of cells incubated with lysosensor. Scale bar, 5 µm. **F.** Quantification of E. after cell background subtraction and normalized by cell area and to Dd wt intensity. **G.** Representative images of cells incubated with DQgreen-BSA. Scale bar, 5 µm. **H.** Quantification of G. after cell background subtraction and normalized by cell area and to Dd wt intensity. Negative values were plotted as 0%. (n=3; N≥3, *p≤0.05, **p≤0.01, ***p≤0.005, ****p≤0.0001, one-way ANOVA - Dunnett test). I-L. BV-2 wt untreated of treated with 1mM of MβCD or 2mM of HβCD were incubated with lysotracker red or DQgreen-BSA for 3h and imaged live by HC microscopy. **I.** Representative images of cells incubated with lysotracker red. Scale bar, 10 µm. **J.** Quantification of I. normalized by cell area. **K.** Representative images of cells incubated with DQgreen-BSA. Scale bar, 10 µm. **L.** Quantification of K. normalized by cell area.

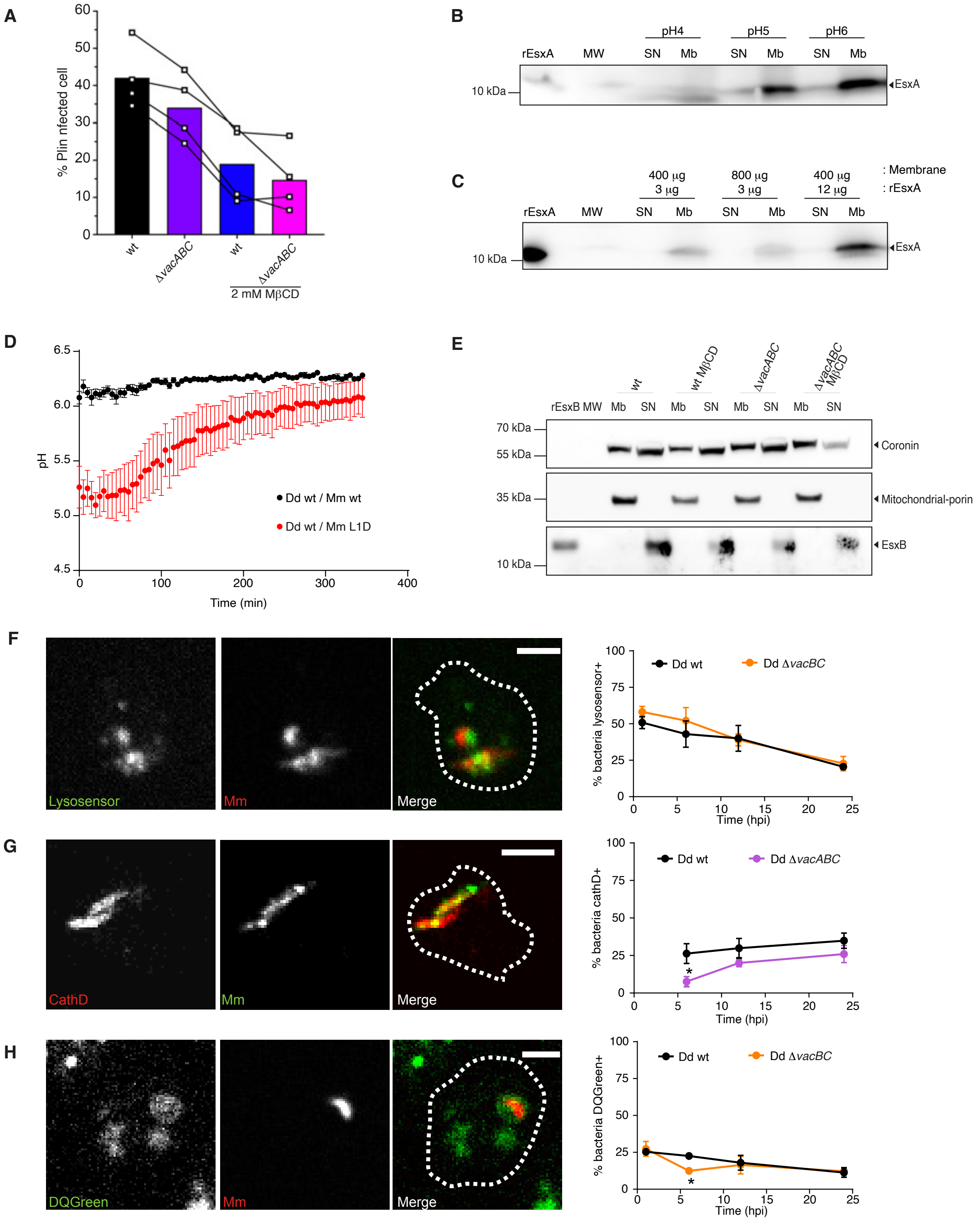

Figure S7: EsxA binds membranes at pH 6, the pH measured in the MCV lumen.

A. Wt and Δ*vacABC* cells expressing mCherry-Plin were infected with GFP-expressing Mm wt and treated or not with 2 mM of MβCD, imaged live by HC microscopy at 24 hpi. Plin recruitment around Mm was quantified (mean + individual replicates, N=4). **B-C**. Recombinant EsxA (rEsxA) was incubated with

purified membranes of wt cells at different pH (**B.**) or membrane to peptide ratios (**C.**), before separation in supernatant (SN) and pellet (Mb-membrane) fractions. Identical protein amounts were loaded and immunoblotted with the indicated antibody. **D.** Wt cells were infected with Mm or Mm-L1D labelled with FITC (pH sensitive) and TRITC (pH insensitive). The ratio of the two fluorescence intensities was monitored (mean ± sem, N=2, n≥3). **E.** Recombinant EsxB (rEsxB) was incubated with post-nuclear supernatant (PNS) of wt or Δ*vacABC* cells, treated or not with 10 mM of MβCD. The PNS was separated into supernatant (SN-cytosol) and pellet (Mb-membrane) fractions. Identical protein amounts were loaded and immunoblotted with the indicated antibodies. **F-H.** Wt and Δ*vacBC* or Δ*vacABC* cells infected with mCherry-expressing Mm wt imaged at the indicated time points after incubation with Lysosensor Green (**F.**) for 10 minutes, immunostained for CathD (**G.**) or after incubation with DQgreen-BSA (**H.**) for 1 hour before imaging. Representative images at 12 hpi are shown on the left. Scale bar, 5 µm. The proportion of marker-positive bacteria for each strain and time point is shown on the right (mean ± s.e.m., N=3, n≥150 MCVs, *p≤0.05, two-way ANOVA - Fisher's _SD).

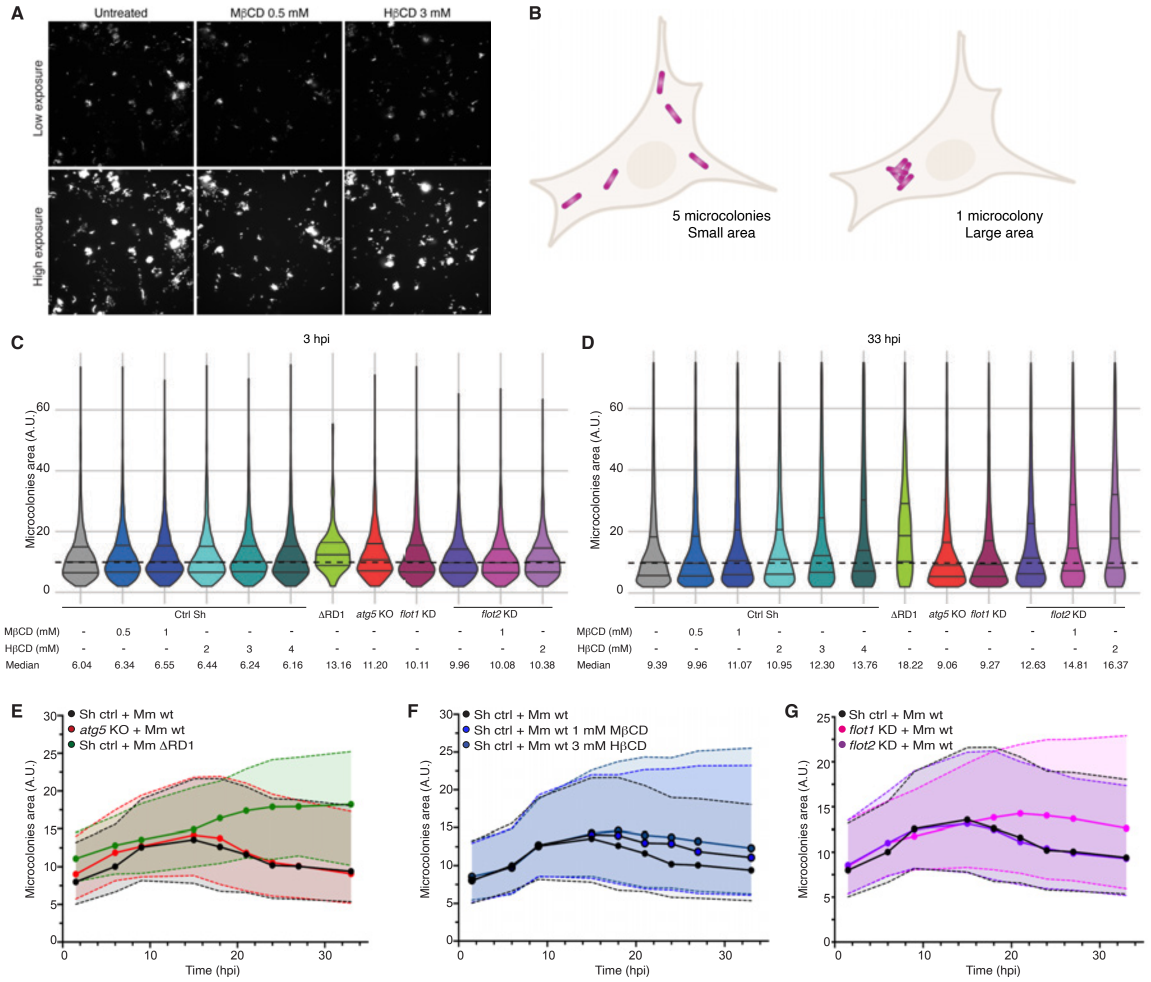

Figure S8: Disruption of membrane microdomains increases Mm microcolonies area in BV-2 cells. A-G. BV-2 cells were infected with GFP-expressing Mm wt and monitored by HC microscopy for 48 hours. **A.** Representative images of bacteria at high or low brightness and contrast. **B.** Scheme illustrating the microcolony morphology. **C-D.** Violin plots with median and quantiles of one representative experiment of the distribution of microcolony area at 3 hpi (**C.**) and 33 hpi (**D.**) . The dashed line represents the median of the control condition, BV-2 wt cells infected with Mm wt. **E-G.** Medians of microcolony area of C-D. are plotted as a time course for each condition and surrounded by the lower and higher quantiles.

Movie S1: Time-lapse movie of wt cells stained with lysosensor and treated with LLOMe

Movie S2: Time-lapse movie of Δ*vacABC* cells stained with lysosensor and treated with LLOMe.

Supplementary Table 1. List of *D. discoideum*, BV-2 and *M. marinum* strains used in this study

Strain/Plasmid	Relevant characteristics	Source/Reference
D. discoideum		
Ax2(Ka)	Wild-type	
Ax2(Ka) ΔvacA knock-out strain		(Bosmani et al., 2020) (38)
Ax2(Ka) ΔvacB knock-out strain		(Bosmani et al., 2020) (38)
Ax2(Ka) ΔvacC knock-out strain		(Bosmani et al., 2020) (38)
Ax2(Ka) ΔvacBΔvacC knock-out strain		(Bosmani et al., 2020) (38)
Ax2(Ka) ΔvacAΔvacBΔVacC knock-out strain		(Bosmani et al., 2020) (38)
Ax2(Ka) VacA-GFP knock-in strain		(Bosmani et al., 2020) (38)
Ax2(Ka) VacB-GFP knock-in strain		(Bosmani et al., 2020) (38)
Ax2(Ka) VacC-GFP knock-in strain		(Bosmani et al., 2020) (38)
Ax2(Ka) and Ax2(ka) ΔvacBC knock-out GFP-VPS32	pDM1513-Vps32: KI in the Act5 locus	This study
Ax2(Ka) and Ax2(ka) ΔvacABC knock-out mCherry-Plin	pDM1514-Plin: KI in the Act5 locus	This study
Ax2(ka) GFP-D4H*	pDM1513-D4H*: KI in the Act5 locus	This study
Ax2(Ka) VacC-GFP knock-in mScarlett-D4H*	pPI419-D4H*: KI in the Act5 locus	This study
BV-2 cells		
BV-2 wt		
BV-2 Atg5 KO		Anthony Orvedahl (Orvedahl et al., 2023) (79)
BV-2 Sh ctrl	pLKO.1-ShC002 (Provided by Mario Tschan)	This study
BV-2 Flot1-KD	pLKO.1- Mouse shFlot1_1548 (Provided by Mario Tschan)	This study
BV-2 Flot2-KD	pLKO.1- Mouse shFlot2_1157 (Provided by Mario Tschan)	This study
BV-2 wt Flot2-GFP	Lentiviral construct: pLenti-Flot2-GFP	This study
BV-2 wt GFP-D4H*	Lentiviral construct: pLenti-GFP-D4H*	This study
BV-2 wt YFP-Gal3	Retroviral construct : M6P-Blast-YFP-Gal3 (Provided by Felix Randow)	This study
Mycobacteria strains		

M. marinum M strain	Wild-type	L. Ramakrishnan (Washington University)
M. marinum ΔRD1		L. Ramakrishnan (Washington University)
M. marinum L1D		L. Ramakrishnan (Washington University)
M. smegmatis		G. Griffiths (EMBL, Heidelberg, Germany)
Mycobacteria plasmids		
pCherry10	mCherry under control of the G13 promoter, Hyg^r	(Carroll et al., 2010) (80)
pMSP12::DsRed/GFP	DsRed/GFP under control of the MSP promoter, Kan^r	(Cosma et al., 2004) (81)
pMV306::*lux*	bacterial luciferase under control of the G13 promoter, Kan^r	(Andreu et al., 2010) (82)
pTEC18	EBFP2 under control of the MSP promoter, Hyg^r	(Takaki et al., 2013) (83)

Supplementary Table 2. List of oligos used in this study, F: forward, R: reverse

**Oligonucleotide/Use**	**Sequence 5'-3'**
qPCR	
gapdh-F	GGTTGTCCCAATTGGTATTAATGG
gapdh-R	CCGTGGGTTGAATCATATTTGAAC
vacA-F	CATTGGCAGATAACAAATCAGCATTGG
vacA-R	ATCATTGGTGGCTTGACCTGGTTTAAT
vacB-F	GAAATTAGATATCTTGGCTCAGTTGCAAA
vacB-R	ATTACCACTTTCACTAGTATCTTCAACCAT
vacC-F	TTAAAGTTGTCGAACAAATAGGTAGTC
vacC-R	CCATCTTTTGGTGTTTGAATAATTTCAAG
Flot1-F	GAGAGTGTGGAATTGCGACTG
Flot1-R	CAGGTTACTCTTCAGCGTCTG
Flot2-F	TGAAGATCATGACGGAGAAGG
Flot2-R	GGTCTCGGTCCTGATAAATCTG
gapdh-F (BV-2)	CCATTTGCAGTGGCAAAGTGG
gapdh-R (BV-2)	GAATTTGCCGTGAGTGGAGTC

Data S1: Raw data.
Raw data for the figures are organized into separate folders. Each folder contains the Prism file for all panels of the corresponding figure and, when applicable, the associated raw Western blot.

REFERENCES

1. World Health Organization, Global TB Rep. 2023 (2023); https://who.int/teams/global-programme-on-tuberculosis-and-lung-health/tb-reports/global-tuberculosis-report-2023.

2. D. G. Russell, *Mycobacterium tuberculosis*: Here today, and here tomorrow. *Nat. Rev. Mol. Cell Biol.* **2**, 569–578 (2001).

3. D. G. Russell, Who puts the tubercle in tuberculosis? *Nat. Rev. Microbiol.* **5**, 39–47 (2007).

4. T. P. Stinear, T. Seemann, P. F. Harrison, G. A. Jenkin, J. K. Davies, P. D. R. Johnson, Z. Abdellah, C. Arrowsmith, T. Chillingworth, C. Churcher, K. Clarke, A. Cronin, P. Davis, I. Goodhead, N. Holroyd, K. Jagels, A. Lord, S. Moule, K. Mungall, H. Norbertczak, M. A. Quail, E. Rabbinowitsch, D. Walker, B. White, S. Whitehead, P. L. C. Small, R. Brosch, L. Ramakrishnan, M. A. Fischbach, J. Parkhill, S. T. Cole, Insights from the complete genome sequence of *Mycobacterium marinum* on the evolution of *Mycobacterium tuberculosis*. *Genome Res.* **18**, 729–741 (2008).

5. D. M. Tobin, L. Ramakrishnan, Comparative pathogenesis of *Mycobacterium marinum* and *Mycobacterium tuberculosis*. *Cell. Microbiol.* **10**, 1027–1039 (2008).

6. J. Boulais, M. Trost, C. R. Landry, R. Dieckmann, E. D. Levy, T. Soldati, S. W. Michnick, P. Thibault, M. Desjardins, Molecular characterization of the evolution of phagosomes. *Mol. Syst. Biol.* **6**, 423 (2010).

7. J. M. Solomon, G. S. Leung, R. R. Isberg, Intracellular replication of *Mycobacterium marinum* within *Dictyostelium discoideum*: Efficient replication in the absence of host coronin. *Infect. Immun.* **71**, 3578–3586 (2003).

8. M. Hagedorn, T. Soldati, Flotillin and RacH modulate the intracellular immunity of *Dictyostelium* to *Mycobacterium marinum* infection. *Cell. Microbiol.* **9**, 2716–2733 (2007).

9. P. Cosson, T. Soldati, Eat, kill or die: When amoeba meets bacteria. *Curr. Opin. Microbiol.* **11**, 271–276 (2008).

10. L. Eichinger, J. A. Pachebat, G. Glöckner, M.-A. Rajandream, R. Sucgang, M. Berriman, J. Song, R. Olsen, K. Szafranski, Q. Xu, B. Tunggal, S. Kummerfeld, M. Madera, B. A. Konfortov, F. Rivero, A. T. Bankier, R. Lehmann, N. Hamlin, R. Davies, P. Gaudet, P. Fey, K. Pilcher, G. Chen, D. Saunders, E. Sodergren, P. Davis, A. Kerhornou, X. Nie, N. Hall, C. Anjard, L. Hemphill, N. Bason, P. Farbrother, B. Desany, E. Just, T. Morio, R. Rost, C. Churcher, J. Cooper, S. Haydock, N. van Driessche, A. Cronin, I. Goodhead, D. Muzny, T. Mourier, A. Pain, M. Lu, D. Harper, R. Lindsay, H. Hauser, K. James, M. Quiles, M. M. Babu, T. Saito, C. Buchrieser, A. Wardroper, M. Felder, M. Thangavelu, D. Johnson, A. Knights, H. Loulseged, K. Mungall, K. Oliver, C. Price, M. A. Quail, H. Urushihara, J. Hernandez, E. Rabbinowitsch, D. Steffen, M. Sanders, J. Ma, Y. Kohara, S. Sharp, M. Simmonds, S. Spiegler, A. Tivey, S. Sugano, B. White, D. Walker, J. Woodward, T. Winckler, Y. Tanaka, G. Shaulsky, M. Schleicher, G. Weinstock, A. Rosenthal, E. C. Cox, R. L. Chisholm, R. Gibbs, W. F. Loomis, M. Platzer, R. R. Kay, J. Williams, P. H. Dear, A. A. Noegel, B. Barrell, A. Kuspa, The genome of the social amoeba *Dictyostelium discoideum*. *Nature* **435**, 43–57 (2005).

11. J. D. Dunn, C. Bosmani, C. Barisch, L. Raykov, L. H. Lefrançois, E. Cardenal-Muñoz, A. T. López-Jiménez, T. Soldati, Eat prey, live: *Dictyostelium discoideum* as a model for cell-autonomous defenses. *Front. Immunol.* **8**, 1906 (2018).

12. S. Guallar-Garrido, T. Soldati, Exploring host–pathogen interactions in the *Dictyostelium discoideum–Mycobacterium marinum* infection model of tuberculosis. *Dis. Model Mech.* **17**, dmm050698 (2024).

13. P. Cosson, W. C. Lima, Intracellular killing of bacteria: Is *Dictyostelium* a model macrophage or an alien? *Cell. Microbiol.* **16**, 816–823 (2014).

14. C. Barisch, V. Kalinina, L. H. Lefrancois, J. Appiah, T. Soldati, Think zinc: Role of zinc poisoning in the intraphagosomal killing of bacteria by the amoeba *Dictyostelium*. bioRxiv 356949 [Preprint] (2018). https://biorxiv.org/content/10.1101/356949v1.full.

15. N. Hanna, H. Koliwer-Brandl, L. H. Lefrançois, V. Kalinina, E. Cardenal-Muñoz, J. Appiah, F. Leuba, A. Gueho, H. Hilbi, T. Soldati, C. Barisch, Zn^{2+} intoxication of *Mycobacterium*

marinum during *Dictyostelium discoideum* infection is counteracted by induction of the pathogen Zn^{2+} exporter CtpC. *MBio* **12**, e01313–e01320 (2021).

16. K. N. Lewis, R. Liao, K. M. Guinn, M. J. Hickey, S. Smith, M. A. Behr, D. R. Sherman, Deletion of RD1 from *Mycobacterium tuberculosis* mimics bacille Calmette-Guérin attenuation. *J. Infect. Dis.* **187**, 117–123 (2003).

17. L. Gao, S. Guo, B. McLaughlin, H. Morisaki, J. N. Engel, E. J. Brown, A mycobacterial virulence gene cluster extending RD1 is required for cytolysis, bacterial spreading and ESAT-6 secretion. *Mol. Microbiol.* **53**, 1677–1693 (2004).

18. E. Cardenal-Muñoz, C. Barisch, L. H. Lefrançois, A. T. López-Jiménez, T. Soldati, When Dicty met Myco, a (not so) romantic story about one amoeba and its intracellular pathogen. *Front. Cell. Infect. Microbiol.* **7**, 529 (2018).

19. E. Cardenal-Muñoz, S. Arafah, A. T. López-Jiménez, S. Kicka, A. Falaise, F. Bach, O. Schaad, J. S. King, M. Hagedorn, T. Soldati, *Mycobacterium marinum* antagonistically induces an autophagic response while repressing the autophagic flux in a TORC1- and ESX-1-dependent manner. *PLOS Pathog.* **13**, e1006344 (2017).

20. A. T. López-Jiménez, E. Cardenal-Muñoz, F. Leuba, L. Gerstenmaier, C. Barisch, M. Hagedorn, J. S. King, T. Soldati, The ESCRT and autophagy machineries cooperate to repair ESX-1-dependent damage at the *Mycobacterium*-containing vacuole but have opposite impact on containing the infection. *PLOS Pathog.* **14**, e1007501 (2018).

21. L. Raykov, M. Mottet, J. Nitschke, T. Soldati, A TRAF-like E3 ubiquitin ligase TrafE coordinates ESCRT and autophagy in endolysosomal damage response and cell-autonomous immunity to *Mycobacterium marinum*. *eLife* **12**, e85727 (2023).

22. E. M. Bernard, A. Fearns, C. Bussi, P. Santucci, C. J. Peddie, R. J. Lai, L. M. Collinson, M. G. Gutierrez, *M. tuberculosis* infection of human iPSDM reveals complex membrane dynamics during xenophagy evasion. *J. Cell Sci.* **134**, jcs252973 (2020).

23. J. Augenstreich, A. Arbues, R. Simeone, E. Haanappel, A. Wegener, F. Sayes, F. Le Chevalier, C. Chalut, W. Malaga, C. Guilhot, R. Brosch, C. Astarie-Dequeker, ESX-1 and phthiocerol dimycocerosates of *Mycobacterium tuberculosis* act in concert to cause phagosomal rupture and host cell apoptosis. *Cell. Microbiol.* **19**, e12726 (2017).

24. C. Cambier, S. Banik, J. A. Buonomo, C. Bertozzi, Spreading of a virulence lipid into host membranes promotes mycobacterial pathogenesis. bioRxiv 845081 [Preprint] (2019). https://doi.org/10.1101/845081.

25. T. R. Lerner, C. J. Queval, A. Fearns, U. Repnik, G. Griffiths, M. G. Gutierrez, Phthiocerol dimycocerosates promote access to the cytosol and intracellular burden of *Mycobacterium tuberculosis* in lymphatic endothelial cells. *BMC Biol.* **16**, 1 (2018).

26. J.-F. Dermine, S. Duclos, J. Garin, F. St-Louis, S. Rea, R. G. Parton, M. Desjardins, Flotillin-1-enriched lipid raft domains accumulate on maturing phagosomes. *J. Biol. Chem.* **276**, 18507–18512 (2001).

27. I. C. Morrow, R. G. Parton, Flotillins and the PHB domain protein family: Rafts, worms and anaesthetics: Flotillins and the PHB family. *Traffic* **6**, 725–740 (2005).

28. G. P. Otto, B. J. Nichols, The roles of flotillin microdomains—Endocytosis and beyond. *J. Cell Sci.* **124**, 3933–3940 (2011).

29. Z. Fu, R. MacKinnon, Structure of the flotillin complex in a native membrane environment. *Proc. Natl. Acad. Sci. U.S.A.* **121**, e2409334121 (2024).

30. D. P. Corkery, A. Nadeem, K. M. Aung, A. Hassan, T. Liu, R. Cervantes-Rivera, A. H. Lystad, H. Wang, K. Persson, A. Puhar, A. Simonsen, B. E. Uhlin, S. N. Wai, Y.-W. Wu, *Vibrio cholerae* cytotoxin MakA induces noncanonical autophagy resulting in the spatial inhibition of canonical autophagy. *J. Cell Sci.* **134**, jcs.252015 (2020).

31. X. Jia, A. Knyazeva, Y. Zhang, S. Castro-Gonzalez, S. Nakamura, L.-A. Carlson, T. Yoshimori, D. P. Corkery, Y.-W. Wu, *V. cholerae* MakA is a cholesterol-binding pore-forming toxin that induces non-canonical autophagy. *J. Cell Biol.* **221**, e202206040 (2022).

32. D. A. Portnoy, V. Auerbuch, I. J. Glomski, The cell biology of *Listeria monocytogenes* infection. *J. Cell Biol.* **158**, 409–414 (2002).

33. B. Arellano-Reynoso, N. Lapaque, S. Salcedo, G. Briones, A. E. Ciocchini, R. Ugalde, E. Moreno, I. Moriyón, J.-P. Gorvel, Cyclic β-1,2-glucan is a *Brucella* virulence factor required for intracellular survival. *Nat. Immunol.* **6**, 618–625 (2005).

34. J. T. Korhonen, M. Puolakkainen, R. Häivälä, T. Penttilä, A. Haveri, E. Markkula, R. Lahesmaa, Flotillin-1 (Reggie-2) contributes to *Chlamydia pneumoniae* growth and is associated with bacterial inclusion. *Infect. Immun.* **80**, 1072–1078 (2012).

35. Q. Xiong, M. Lin, W. Huang, Y. Rikihisa, Infection by *Anaplasma phagocytophilum* requires recruitment of low-density lipoprotein cholesterol by flotillins. *MBio* **10**, e02783-18 (2019).

36. N. Jenne, R. Rauchenberger, U. Hacker, T. Kast, M. Maniak, Targeted gene disruption reveals a role for vacuolin B in the late endocytic pathway and exocytosis. *J. Cell Sci.* **111**, 61–70 (1998).

37. D. Wienke, A. Drengk, C. Schmauch, N. Jenne, M. Maniak, Vacuolin, a flotillin/reggie-related protein from *Dictyostelium* oligomerizes for endosome association. *Eur. J. Cell Biol.* **85**, 991–1000 (2006).

38. C. Bosmani, F. Leuba, N. Hanna, F. Bach, F. Burdet, M. Pagni, M. Hagedorn, T. Soldati, Vacuolins and myosin VII are required for phagocytic uptake and phagosomal membrane recycling in *Dictyostelium discoideum*. *J. Cell Sci.* **133**, jcs242974 (2020).

39. N. Hanna, F. Burdet, A. Melotti, C. Bosmani, S. Kicka, H. Hilbi, P. Cosson, M. Pagni, T. Soldati, Time-resolved RNA-seq profiling of the infection of *Dictyostelium discoideum* by *Mycobacterium marinum* reveals an integrated host response to damage and stress. bioRxiv 590810 [Preprint] (2019). https://doi.org/10.1101/590810.

40. M. Stajdohar, R. D. Rosengarten, J. Kokosar, L. Jeran, D. Blenkus, G. Shaulsky, B. Zupan, dictyExpress: A web-based platform for sequence data management and analytics in *Dictyostelium* and beyond. *BMC Bioinformatics* **18**, 291 (2017).

41. O. Lamrabet, T. Jauslin, W. C. Lima, M. Leippe, P. Cosson, The multifarious lysozyme arsenal of *Dictyostelium discoideum*. *Dev. Comp. Immunol.* **107**, 103645 (2020).

42. M. Marek, V. Vincenzetti, S. G. Martin, Sterol biosensor reveals LAM-family Ltc1-dependent sterol flow to endosomes upon Arp2/3 inhibition. *J. Cell Biol.* **219**, e202001147 (2020).

43. K.-P. Janssen, R. Rost, L. Eichinger, M. Schleicher, Characterization of CD36/LIMPII homologues in *Dictyostelium discoideum*. *J. Biol. Chem.* **276**, 38899–38910 (2001).

44. E. Blasi, R. Barluzzi, V. Bocchini, R. Mazzolla, F. Bistoni, Immortalization of murine microglial cells by a v-raf/v-myc carrying retrovirus. *J. Neuroimmunol.* **27**, 229–237 (1990).

45. L. H. Lefrançois, J. Nitschke, H. Wu, G. Panis, J. Prados, R. E. Butler, T. A. Mendum, N. Hanna, G. R. Stewart, T. Soldati, Temporal genome-wide fitness analysis of *Mycobacterium marinum* during infection reveals the genetic requirement for virulence and survival in amoebae and microglial cells. *mSystems* **9**, e0132623 (2024).

46. V. Trofimov, S. Kicka, S. Mucaria, N. Hanna, F. Ramon-Olayo, L. V.-G. Del Peral, J. Lelièvre, L. Ballell, L. Scapozza, G. S. Besra, J. A. G. Cox, T. Soldati, Antimycobacterial drug discovery using Mycobacteria-infected amoebae identifies anti-infectives and new molecular targets. *Sci. Rep.* **8**, 3939 (2018).

47. A. Goujon, A. Colom, K. Straková, V. Mercier, D. Mahecic, S. Manley, N. Sakai, A. Roux, S. Matile, Mechanosensitive fluorescent probes to image membrane tension in mitochondria, endoplasmic reticulum, and lysosomes. *J. Am. Chem. Soc.* **141**, 3380–3384 (2019).

48. H. F. Clark, C. C. Shepard, Effect of environmental temperatures on infection with *Mycobacterium marinum* (balnei) of mice and a number of poikilothermic species. *J. Bacteriol.* **86**, 1057–1069 (1963).

49. P. Fey, A. S. Kowal, P. Gaudet, K. E. Pilcher, R. L. Chisholm, Protocols for growth and development of *Dictyostelium discoideum*. *Nat. Protoc.* **2**, 1307–1316 (2007).

50. M. Hagedorn, K. H. Rohde, D. G. Russell, T. Soldati, Infection by tubercular mycobacteria is spread by nonlytic ejection from their amoeba hosts. *Science* **323**, 1729–1733 (2009).

51. G. R. Golovkine, A. W. Roberts, H. M. Morrison, R. Rivera-Lugo, R. M. McCall, H. Nilsson, N. E. Garelis, T. Repasy, M. Cronce, J. Budzik, E. Van Dis, L. M. Popov, G. Mitchell, R. Zalpuri, D. Jorgens, J. S. Cox, Autophagy restricts *Mycobacterium tuberculosis* during acute infection in mice. *Nat. Microbiol.* **8**, 819–832 (2023).

52. F. Wang, R. Peters, J. Jia, M. Mudd, M. Salemi, L. Allers, R. Javed, T. L. A. Duque, M. A. Paddar, E. S. Trosdal, B. Phinney, V. Deretic, ATG5 provides host protection acting as a switch in the atg8ylation cascade between autophagy and secretion. *Dev. Cell* **58**, 866–884.e8 (2023).

53. E. Mittal, M. L. Skowyra, G. Uwase, E. Tinaztepe, A. Mehra, S. Köster, P. I. Hanson, J. A. Philips, *Mycobacterium tuberculosis* type VII secretion system effectors differentially impact the ESCRT endomembrane damage response. *MBio* **9**, e01765-18–e01765-18 (2018).

54. C. Bussi, A. Mangiarotti, C. Vanhille-Campos, B. Aylan, E. Pellegrino, N. Athanasiadi, A. Fearns, A. Rodgers, T. M. Franzmann, A. Šarić, R. Dimova, M. G. Gutierrez, Stress granules plug and stabilize damaged endolysosomal membranes. *Nature* **623**, 1062–1069 (2023).

55. M. Vietri, M. Radulovic, H. Stenmark, The many functions of ESCRTs. *Nat. Rev. Mol. Cell Biol.* **21**, 25–42 (2020).

56. C. M. Buckley, V. L. Heath, A. Guého, C. Bosmani, P. Knobloch, P. Sikakana, N. Personnic, S. K. Dove, R. H. Michell, R. Meier, H. Hilbi, T. Soldati, R. H. Insall, J. S. King, PIKfyve/Fab1 is required for efficient V-ATPase and hydrolase delivery to phagosomes, phagosomal killing, and restriction of Legionella infection. *PLOS Pathog.* **15**, e1007551 (2019).

57. C. Barisch, P. Paschke, M. Hagedorn, M. Maniak, T. Soldati, Lipid droplet dynamics at early stages of *Mycobacterium marinum* infection in *Dictyostelium*: Lipid droplets in mycobacterium infection. *Cell. Microbiol.* **17**, 1332–1349 (2015).

58. M. I. de Jonge, G. Pehau-Arnaudet, M. M. Fretz, F. Romain, D. Bottai, P. Brodin, N. Honore, G. Marchal, W. Jiskoot, P. England, S. T. Cole, R. Brosch, ESAT-6 from *Mycobacterium tuberculosis* dissociates from its putative chaperone CFP-10 under acidic conditions and exhibits membrane-lysing activity. *J. Bacteriol.* **189**, 6028–6034 (2007).

59. D. Koiri, M. Nandi, P. M. A. Hameem, J. B. Aher, A. Kumar, A. Behura, G. Meher, V. Choudhary, S. Choubey, M. Saleem, Real-time visualization reveals *Mycobacterium tuberculosis* ESAT-6 disrupts phagosome-like compartment via fibril-mediated vesiculation. *Cell Rep.* **44**, 115328 (2025).

60. S. Ray, S. Vazquez Reyes, C. Xiao, J. Sun, Effects of membrane lipid composition on *Mycobacterium tuberculosis* EsxA membrane insertion: A dual play of fluidity and charge. *Tuberculosis* **118**, 101854 (2019).

61. C. Astarie-Dequeker, L. L. Guyader, W. Malaga, F.-K. Seaphanh, C. Chalut, A. Lopez, C. Guilhot, Phthiocerol dimycocerosates of *M. tuberculosis* participate in macrophage invasion by inducing changes in the organization of plasma membrane lipids. *PLOS Pathogens* **5**, e1000289 (2009).

62. J. Augenstreich, E. Haanappel, G. Ferré, G. Czaplicki, F. Jolibois, N. Destainville, C. Guilhot, A. Milon, C. Astarie-Dequeker, M. Chavent, The conical shape of DIM lipids promotes *Mycobacterium tuberculosis* infection of macrophages. *Proc. Natl. Acad. Sci. U.S.A.* **116**, 25649–25658 (2019).

63. C. Barisch, T. Soldati, Breaking fat! How mycobacteria and other intracellular pathogens manipulate host lipid droplets. *Biochimie* **141**, 54–61 (2017).

64. M. Foulon, S. A. Listian, T. Soldati, C. Barisch, "Conserved mechanisms drive host-lipid access, import, and utilization in *Mycobacterium tuberculosis* and *M. marinum*," in *Biology of Mycobacterial Lipids* (Elsevier, 2022), pp. 133–161; https://linkinghub.elsevier.com/retrieve/pii/B9780323919487000117.

65. A. Guého, C. Bosmani, J. Nitschke, T. Soldati, Proteomic characterization of the *Mycobacterium marinum*-containing vacuole in *Dictyostelium discoideum*. bioRxiv 592717 [Preprint] (2019). https://doi.org/10.1101/592717.

66. A. Machelart, G. Salzano, X. Li, A. Demars, A.-S. Debrie, M. Menendez-Miranda, E. Pancani, S. Jouny, E. Hoffmann, N. Deboosere, I. Belhaouane, C. Rouanet, S. Simar, S. Talahari, V. Giannini, B. Villemagne, M. Flipo, R. Brosch, F. Nesslany, B. Deprez, E. Muraille, C. Locht, A. R. Baulard, N. Willand, L. Majlessi, R. Gref, P. Brodin, Intrinsic antibacterial activity of nanoparticles made of β-cyclodextrins potentiates their effect as drug nanocarriers against tuberculosis. *ACS Nano* **13**, 3992–4007 (2019).

67. T. T. B. Tram, L. C. Garner, L. N. H. Thai, L. T. H. Nhat, D. D. A. Thu, H. D. T. Nghia, L. H. Van, G. E. Thwaites, V. T. N. Ha, P. Klenerman, N. T. T. Thuong, Single-cell profiling of blood and cerebrospinal fluid in tuberculous meningitis. *J. Immunol.* **214**, 2894–2905 (2025).

68. J. D. Leon, G. Jiang, Y. Ma, E. Rubin, S. Fortune, J. Sun, *Mycobacterium tuberculosis* ESAT-6 exhibits a unique membrane-interacting activity that is not found in its ortholog from non-pathogenic *Mycobacterium smegmatis*. *J. Biol. Chem.* **287**, 44184–44191 (2012).

69. J. Augenstreich, V. Briken, Host cell targets of released lipid and secreted protein effectors of *Mycobacterium tuberculosis*. *Front. Cell. Infect. Microbiol.* **10**, 595029 (2020).

70. J. Aguilera, C. B. Karki, L. Li, S. Vazquez Reyes, I. Estevao, B. I. Grajeda, Q. Zhang, C. D. Arico, H. Ouellet, J. Sun, N α-acetylation of the virulence factor EsxA is required for mycobacterial cytosolic translocation and virulence. *J. Biol. Chem.* **295**, 5785–5794 (2020).

71. C. Cambier, S. M. Banik, J. A. Buonomo, C. R. Bertozzi, Spreading of a mycobacterial cell-surface lipid into host epithelial membranes promotes infectivity. *eLife* **9**, e60648 (2020).

72. Y. Luo, C.-C. Huang, N. C. Howard, X. Wang, Q. Liu, X. Li, J. Zhu, T. Amariuta, S. Asgari, K. Ishigaki, R. Calderon, S. Raman, A. K. Ramnarine, J. A. Mayfield, D. B. Moody, L. Lecca, S. M. Fortune, M. B. Murray, S. Raychaudhuri, Paired analysis of host and pathogen genomes identifies determinants of human tuberculosis. *Nat. Commun.* **15**, 10393 (2024).

73. A. Journet, A. Chapel, S. Jehan, C. Adessi, H. Freeze, G. Klein, J. Garin, Characterization of *Dictyostelium discoideum* cathepsin D. *J. Cell Sci.* **112**, 3833–3843 (1999).

74. E. C. Schwarz, E. M. Neuhaus, C. Kistler, A. W. Henkel, T. Soldati, Dictyostelium myosin IK is involved in the maintenance of cortical tension and affects motility and phagocytosis. *J. Cell Sci.* **113**, 621–633 (2000).

75. M. Hagedorn, E. M. Neuhaus, T. Soldati, "Optimized fixation and immunofluorescence staining methods for *Dictyostelium* cells," in *Dictyostelium Discoideum Protocols* (Humana Press, 2006), vol. 346, pp. 327–338; http://link.springer.com/10.1385/1-59745-144-4:327.

76. S. Arafah, S. Kicka, V. Trofimov, M. Hagedorn, N. Andreu, S. Wiles, B. Robertson, T. Soldati, Setting up and monitoring an infection of *Dictyostelium discoideum* with mycobacteria. *Methods Mol. Biol.* **983**, 403–417 (2013).

77. A. Perret, C. Michard, D. Moreau, T. Soldati, "Tiny organisms, big advantages: Exploring membrane damage responses using *Dictyostelium discoideum* in high throughput single cell analyses," in *Live Biological Imaging Across Scales*, S. Linder, C. Wells, Eds. Wiley book chapter (2026).

78. L. Alibaud, Y. Rombouts, X. Trivelli, A. Burguière, S. L. G. Cirillo, J. D. Cirillo, J.-F. Dubremetz, Y. Guérardel, G. Lutfalla, L. Kremer, *A Mycobacterium marinum* TesA mutant defective for major cell wall-associated lipids is highly attenuated in *Dictyostelium discoideum* and zebrafish embryos. *Mol. Microbiol.* **80**, 919–934 (2011).

79. A. Orvedahl, M. R. McAllaster, A. Sansone, B. F. Dunlap, C. Desai, Y.-T. Wang, D. R. Balce, C. J. Luke, S. Lee, R. C. Orchard, M. N. Artyomov, S. A. Handley, J. G. Doench, G. A. Silverman, H. W. Virgin, Autophagy genes in myeloid cells counteract IFNγ-induced TNF-mediated cell death and fatal TNF-induced shock. *Proc. Natl. Acad. Sci. U.S.A.* **116**, 16497–16506 (2019).

80. P. Carroll, L. J. Schreuder, J. Muwanguzi-Karugaba, S. Wiles, B. D. Robertson, J. Ripoll, T. H. Ward, G. J. Bancroft, U. E. Schaible, T. Parish, Sensitive detection of gene expression in

mycobacteria under replicating and non-replicating conditions using optimized far-red reporters. *PLOS ONE* **5**, e9823 (2010).

81. C. L. Cosma, O. Humbert, L. Ramakrishnan, Superinfecting mycobacteria home to established tuberculous granulomas. *Nat. Immunol.* **5**, 828–835 (2004).

82. N. Andreu, A. Zelmer, T. Fletcher, P. T. Elkington, T. H. Ward, J. Ripoll, T. Parish, G. J. Bancroft, U. Schaible, B. D. Robertson, S. Wiles, Optimisation of bioluminescent reporters for use with mycobacteria. *PLOS ONE* **5**, e10777 (2010).

83. K. Takaki, J. M. Davis, K. Winglee, L. Ramakrishnan, Evaluation of the pathogenesis and treatment of *Mycobacterium marinum* infection in zebrafish. *Nat. Protoc.* **8**, 1114–1124 (2013).
